# Supplementary material for: A mathematical model of H5N1 influenza transmission in US dairy cattle
Source: Nat Commun. 2025 May 8;16:4308. doi: 10.1038/s41467-025-59554-z (PMC12062519; doi:10.1038/s41467-025-59554-z)
Supplement: Supplementary file 1 — Supplementary Information [file 41467_2025_59554_MOESM1_ESM.pdf]

Supplementary Material

CONTENTS

1 US Cattle Populations 2

2 Methods 4
2.1 Model Equations 4
2.2 Model Logic 5
2.3 Reporting an outbreak 5
2.4 Moving cattle between herds. 6
2.5 Epidemiological Parameter Fitting 13

3 Supplementary Results 20
3.1 Additional Results 20
3.2 Sensitivity Analyses 25
3.2.1 Alternate likelihood 25
3.2.2 Real ICVI data 29
3.2.3 Alternate Ascertainment 32
3.2.4 Birth and Death Processes 35

List of Figures 40

List of Tables 41

1 US Cattle Populations

Data on the number of dairy cattle in each of the 48 continental US states is taken from the United States 2022 Census of Agriculture [1]. Table S1 below details the information extracted from the census on the total number of dairy cattle and dairy herds in each US state. It also lists the mean herd size (number of cattle divided by number of herds), however the census does provide more detailed information in the distribution of herd sizes which is used within the model.

Figure S1 plots the mean herd size by US state, ordered left-to-right from the state with the largest total dairy cow population (California, 1,688,202 cows) to the smallest (Rhode Island, 722 cows). Those states that have declared outbreaks as of December 2nd 2024 are shaded in red. The plot demonstrates how the total number of cows per state does not align with mean herd size. For example, Wisconsin has the second largest total number of dairy cows in the country, but has a far smaller mean herd size than California, as its cattle population is distributed across a large number of smaller holdings.

Table S1: The total number of dairy cattle and the number of dairy herds in each of the 48 continental US states (Hawaii and Alaska not included).

State	Total Cattle Population	Total Herds	Mean Herd Size
Alabama	2,225	92	24.18
Arizona	199,279	122	1,633.43
Arkansas	3,820	75	50.93
California	1,688,202	1,117	1,511.37
Colorado	201,750	414	487.32
Connecticut	18,889	145	130.27
Delaware	2,528	41	61.66
Florida	94,069	369	254.93
Georgia	72,830	391	186.27
Idaho	664,479	549	1,210.34
Illinois	74,918	607	123.42
Indiana	183,176	1,521	120.43
Iowa	238,087	1,016	234.34
Kansas	175,670	501	350.64
Kentucky	45,904	1,062	43.22
Louisiana	7,796	74	105.35
Maine	24,836	292	85.05
Maryland	38,290	342	111.96
Massachusetts	9,998	151	66.21
Michigan	436,254	1,481	294.57
Minnesota	459,536	2,185	210.31
Mississippi	6,904	45	153.42
Missouri	63,882	1,704	37.49
Montana	10,841	273	39.71
Nebraska	57,036	300	190.12
Nevada	32,021	48	667.10
New Hampshire	11,451	129	88.77
New Jersey	4,563	95	48.03
New Mexico	279,664	233	1,200.27
New York	631,199	2,783	226.81
North Carolina	36,127	402	89.87
North Dakota	14,191	107	132.63
Ohio	247,743	2,195	112.87
Oklahoma	40,457	298	135.76
Oregon	117,533	516	227.78
Pennsylvania	455,651	4,027	113.15
Rhode Island	722	13	55.54
South Carolina	10,203	153	66.69
South Dakota	188,764	255	740.25
Tennessee	24,525	680	36.07
Texas	639,506	479	1,335.09
Utah	91,505	334	273.97

Vermont	106,632	528	201.95
Virginia	65,218	703	92.77
Washington	255,872	419	610.67
West Virginia	4,268	335	12.74
Wisconsin	1,264,272	6,216	203.39
Wyoming	5,421	157	34.53

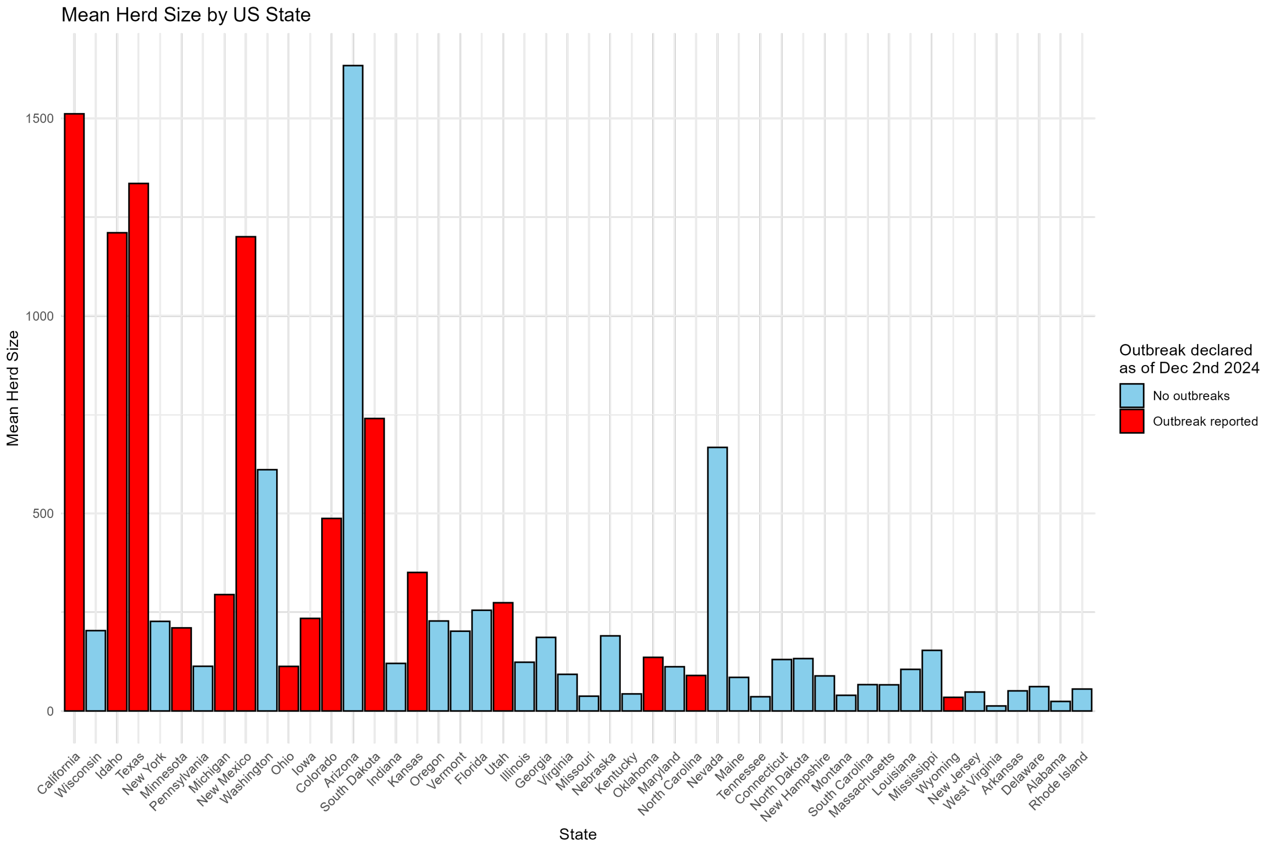

Figure S1: The mean herd size (number of dairy cows divided by number of herds) for each of the 48 continental US states. States are presented, left-to-right, in order of largest total dairy cow population to smallest. States highlighted in red are those that have reported an outbreak of H5N1 in dairy cattle as of December 2nd 2024.

2 Methods

2.1 Model Equations

In this section we provide a more extensive and in-depth explanation of the underlying model mechanics. A metapopulation model of 35,974 herds, modelled as 35,974 separate SEIR frameworks is built, with a total of 9,308,707 dairy cows distributed across these 35,974 herds. Index i refers to the herd, and index j to the US state. Each cow in herd $\{i, j\}$ is compartmentalised within either the susceptible, $S_{i,j}$, exposed, $E_{i,j}$, infected (and infectious), $I_{i,j}$, or recovered, $R_{i,j}$, compartments. Thus, $S_{i,j}$ refers to the susceptible cows in herd i in state j. The basic epidemiological dynamics of the spread of infection within each individual herd can be described by the following series of ODEs:

$$\frac{dS_{i,j}}{dt} = -\beta S_{i,j}\frac{I_{i,j}}{N_{i,j}}, \tag{1}$$

$$\frac{dE_{i,j}}{dt} = \beta S_{i,j}\frac{I_{i,j}}{N_{i,j}} - \sigma E_{i,j}, \tag{2}$$

$$\frac{dI_{i,j}}{dt} = \sigma E_{i,j} - \gamma I_{i,j}, \tag{3}$$

$$\frac{dR_{i,j}}{dt} = \gamma I_{i,j}. \tag{4}$$

All model parameters are presented in Table S2. $N_{i,j}$ is the total number of cows in herd i of state j: $N_{i,j} = S_{i,j} + E_{i,j} + I_{i,j} + R_{i,j}$.

The above equations currently do not allow for any infections to occur across or between herds. Recognising that many neighbouring dairy herds will share workers, grazing areas, equipment, or be exposed to environmental run-off from one-another, we adapt equations (1) and (2) above to allow for herds within the same US state to cause infections between one-another. This transmission occurs at a reduced rate controlled by the parameter α:

$$\frac{dS_{i,j}}{dt} = -\beta S_{i,j}\left(\frac{I_{i,j}}{N_{i,j}} + \alpha\frac{I_{-i,j}}{N_{-i,j}}\right), \tag{5}$$

$$\frac{dE_{i,j}}{dt} = \beta S_{i,j}\left(\frac{I_{i,j}}{N_{i,j}} + \alpha\frac{I_{-i,j}}{N_{-i,j}}\right) - \sigma E_{i,j}, \tag{6}$$

$$\frac{dI_{i,j}}{dt} = \sigma E_{i,j} - \gamma I_{i,j}, \tag{7}$$

$$\frac{dR_{i,j}}{dt} = \gamma I_{i,j}. \tag{8}$$

Here, $I_{-i,j}$ denotes the total number of infected cattle across all herds in state j except for herd i. Likewise, $N_{-i,j}$ denotes the total number of cattle across all herds in state j except for herd i. We note that, under this modeling assumption, there is no difference in the force of infection one herd applies to all other herds in the US state it resides it. Further spatial kernel approaches would require substantial additional information on the precise geo-location of each dairy herd, and the extent to which equipment, workforce, and services is shared between specific herds.

Parameter	Definition
β	Transmission rate.
α	Proportion decrease of transmission rate between cows in different herds, but within the same state.
σ	Incubation rate.
γ	Recovery rate.
A^{asc}	Global ascertainment rate scaling. A model parameter controlling how many infections are reported.

Table S2: Definitions of model parameters.

While early epidemiological reports highlighted that $< 10\%$ of cows on infected herds displayed clinical symptoms [2], virological surveys of these farms identified viral H5N1 RNA within non-clinical animals on infected premises as well [3]. Due to this uncertainty, we do not enforce strict infection carrying capacities into the within-herd model dynamics.

Rather than use the above deterministic series of ODEs, we model the transition between epidemiological states via the stochastic interpretation of equations (5) - (8). We calculate the number of cattle progressing between epidemiological

compartments via binomial distributions, for each timestep dt as:

$$n_{SE} \sim \text{Binomial}\left(S_{i,j},\, 1-\exp\left(-\left(\beta\frac{S_{i,j}I_{i,j}}{N_{i,j}} + \beta S_{i,j}\alpha \sum_{k=1,k\neq i}^{N^{j}_{\text{herds}}} \frac{I_{k,j}}{N_j - N_{k,j}}\right)dt\right)\right), \tag{9}$$

$$n_{EI} \sim \text{Binomial}\left(E_{i,j},\, 1-\exp(-\sigma\, dt)\right), \tag{10}$$

$$n_{IR} \sim \text{Binomial}\left(I_{i,j},\, 1-\exp(-\gamma dt)\right). \tag{11}$$

Here n_{XY} is the number of cattle moved from compartment X to Y (for general X and Y) in a time step of size dt. n_{SE}, n_{EI}, and n_{IR} is calculated for each herd.

2.2 Model Logic

The previous section outlined the underlying epidemiological dynamics dictating the movement of cattle between epidemiological compartments. However, within each timestep of model evaluation, we also calculate multiple other movements between compartments, and construct other model output variables. We probabilistically calculate the movement of cattle between herds and states, as is frequently done within the dairy industry. We also probabilistically calculate whether or not such exports proceed subject to border testing at certain periods of time. We also calculate whether or not each herd will declare an outbreak present at each time point. Algorithm 1 below outlines the mechanical steps taken at each time point in pseudocode. We move cattle between epidemiological compartments following the methods outlined above in equations (9) - (11). We then calculate whether or not each herd reports an outbreak. We then simulate the shipment of cattle between herds for each herd.

Algorithm 1 Model calculations at each timestep.

```
for each time t do
    for each herd i do
        Calculate and move all cows between epidemiological compartments.
    end for

    for each herd i do
        Calculate whether or not herd i reports an outbreak.
    end for

    for each herd i do
        Stochastically decide whether or not any cows will be exported from herd i.
        if cows will be exported then
            Stochastically decide which herd the exported cattle will be exported to.
            if Destination herd is in a different state then
                Simulate the testing of up to 30 random cows in the cohort for H5N1.
                if H5N1 detected then
                    Cancel export.
                else
                    Move cattle from herd i to their same epidemiological compartments in the destination herd.
                end if
            end if
        end if
    end for

end for
```

The entire model code can be seen in the `inst/dust/cows.cpp` file within our associated `cowflu` package. We explain each segment in greater detail in the following subsections.

2.3 Reporting an outbreak

After moving cattle between epidemiological compartments, we then stochastically calculate, for each herd, whether or not that herd will report an outbreak in that time step. If a herd has previously reported an outbreak, we skip this herd - a herd cannot report an outbreak twice. For each herd, we calculate the baseline probability of declaring an outbreak,

ϕ_i, as being dependent on the number of infected cattle in the herd, I_i, and on the proportion of the herd that is infected $\frac{I_i}{N_i}$:

$$\phi_i = \left(\frac{I_i}{(0.7N_i)^{0.95}} + \frac{I_i}{150} \right) A^{\text{asc}} dt, \tag{12}$$

where A^{asc} is a model parameter that we fit, and dt is the size of the timestep. Figure 3A in the main manuscript plots the form of this function for differing values of I_i and N_i. In general, the probability of declaring an outbreak increases as the total number of infected cattle increases, and as the proportion of the herd that is infected increases.

To ensure this probability is always bounded between 0 and 1 for large time steps, we scale ϕ_i via: $P_i^{\text{outbreak}} = 1 - e^{-\phi_i}$. For each time step, for each herd, we draw a random number, X, from a standard Uniform(0,1) distribution, and record a reported outbreak for that herd at that timepoint if $X < P_i^{\text{outbreak}}$.

While different US states are undoubtedly likely to differ in their rates of detection subject to public health staffing or resourcing, fitting individual state level ascertainment scalings would require some degree of observations in all states. The majority of US states have not declared any outbreaks to date. If we were to fit unique A^{asc} values for each state, these fit values would generate posterior distributions very close to 0, to maximise the likelihood function. By assuming one global parameter, we are instead able to identify which states it is reasonable to have observed no outbreaks in, and which it is unreasonable - suggesting under-reporting. For example, Arizona and Rhode Island have both yet to declare any outbreaks of H5N1 in dairy cattle within the state. Our model results in Figure 2B of the main manuscript, however, demonstrate that while that is a very plausible outcome in the case of Rhode Island, that is unlikely to be a true representation of the disease burden in Arizona.

These modeling assumptions were developed in consultation with veterinarian staff, informed by their experiences of the stage at which illnesses in a herd are reported. Assuming a number of cows in a herd are stricken by behavioral changes and reduced milk production, how many cows must exhibit such symptoms before a veterinarian is consulted? Starting from experiences with large holdings of thousands of dairy cattle, the point at which an infection influencing milk production is more likely to be noticed than not, was considered to be approximately 100 infected cattle. At this stage, not only would a sufficiently large number of cattle be identified within the same milking line, but a communicable pathogen is likely to be suspected. Hence, the yellow "50% probability of ascertainment" region of Figure 3A in the main manuscript, is seen to intercept the x-axis at 100 infected cows. Naturally, however, for smaller population herds, an infection passes the point of "more likely to be identified than not" at a smaller number of infected cattle. This point scales with herd population size to a limit such as, in a herd of only 2 cattle, an infection is more likely to be identified than not, as soon as 1 of the 2 cows is infected. Hence, the yellow "50% probability of ascertainment" region of Figure 3A in the main manuscript, is seen to intercept the y-axis at 50% of the herd infected. Note that these values are then further scaled by the fit model parameter A^{asc}.

2.4 Moving cattle between herds.

After calculating which herds report new outbreaks, we simulate the trade of cattle between premises across the country. For each herd, we first ascertain, stochastically, whether or not each herd will, or will not, export cattle this time step. The US Animal Movement Model (USAMM) provides model simulations of the annual movement of dairy cattle between premises [4]. The USAMM is trained on real world Interstate Certificates of Veterinary Inspection (ICVI) data. Whenever cattle are moved across state lines, an ICVI must be issued as record of the movement. These are mostly paper records which were accessed on request by the developers of the USAMM. 1000 stochastic realisations are provided from the USAMM model at `https://mountainscholar.org/items/75fd3841-11c3-4cec-91b5-45d36b8c6b5a`. Each realisation is a simulated annual ledger of all animal movements between premises (including the number of cattle shipped) as based on previous real world exports. It is from this dataset that we infer our probabilities of whether or not a herd will export cattle at any given time step.

We sum the total number of exports from each state, and divide by the total number of herds in that state, to get the mean number of exports from each herd, annually. We divide this value by $\frac{7}{365}$ for the mean weekly number of exports from each herd for each state. This value is then further scaled by the size of the timestep dt. We present these state specific "probabilities of exporting cattle each week" for a herd in Table S3, and in Figure S2.

Table S3: The probability, for each state, that a dairy herd within that state will export dairy cattle in a 7-day period.

State	Weekly probability of a herd exporting cattle
Alabama	0.007901
Arizona	0.087296
Arkansas	0.057525
California	0.298799
Colorado	0.025591
Connecticut	0.020105
Delaware	0.010928
Florida	0.069742
Georgia	0.020383
Idaho	0.045670
Illinois	0.012280
Indiana	0.007776
Iowa	0.033667
Kansas	0.016292
Kentucky	0.012766
Louisiana	0.006318
Maine	0.004024
Maryland	0.017974
Massachusetts	0.008827
Michigan	0.014362
Minnesota	0.019918
Mississippi	0.026062
Missouri	0.003321
Montana	0.008265
Nebraska	0.004062
Nevada	0.017928
New Hampshire	0.005835
New Jersey	0.017042
New Mexico	0.056869
New York	0.046736
North Carolina	0.010130
North Dakota	0.044735
Ohio	0.005976
Oklahoma	0.024752
Oregon	0.011311
Pennsylvania	0.004942
Rhode Island	0.009117
South Carolina	0.035950
South Dakota	0.014929
Tennessee	0.017748
Texas	0.076588
Utah	0.019551
Vermont	0.015776
Virginia	0.009487
Washington	0.039005
West Virginia	0.009223
Wisconsin	0.011569
Wyoming	0.003867

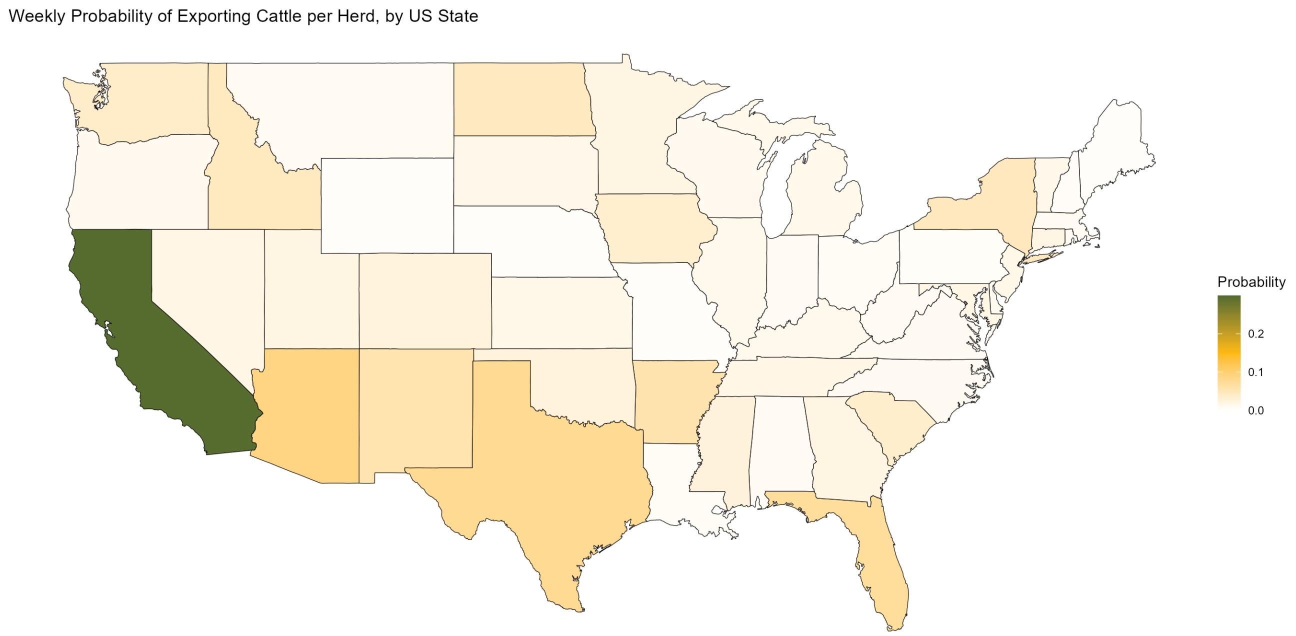

Figure S2: A heatmap of P_k^{export}, the weekly probability of a herd exporting cattle, as given in Table S3. Greater values indicate states which export cattle more frequently.

The USAMM simulation data captures only minor seasonal patterns by quarter (Figure S3), hence we make the simplifying assumption to keep a fixed value for all export probabilities across the year.

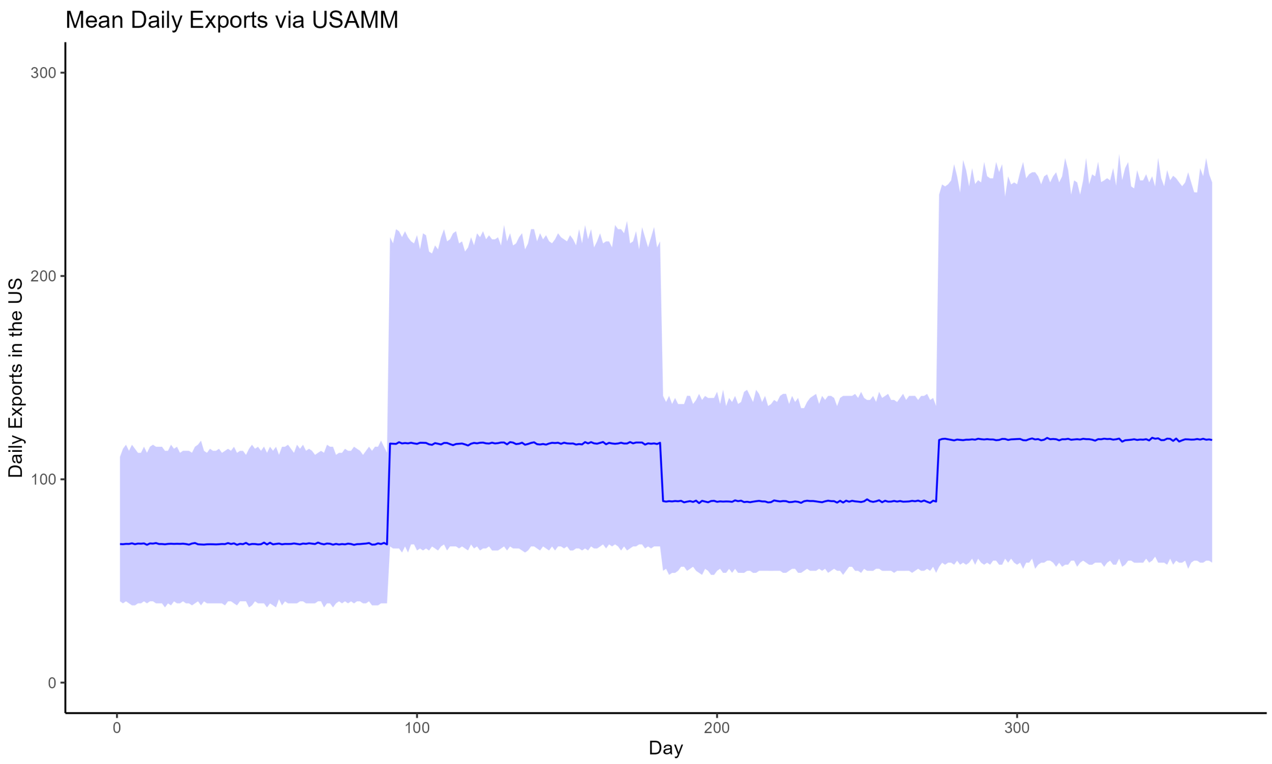

Figure S3: The number of daily exports of dairy cattle in the United States taken from USAMM simulation data [4]. The thick blue line depicts the mean across all simulations and the shaded region the 95% CrI.

At each time step of the model, for each herd, we draw a random number from the standard uniform distribution, $X \sim$ Uniform(0,1). If X is less than the above values, that herd is denoted as exporting cattle in that timestep. We then calculate how many cattle from the herd will be exported. The USAMM simulated data also includes simulated numbers of cattle exported, and the size of the herd of origin. From this we can average, across all simulations and all exports of cattle, the mean proportion of a herd that is exported for each US state. These values will differ as some states will prioritise breeding and selling of milk cattle, while others will import more calves for milk stock. We notate this proportion as $P_j^{\text{export size}}$, the average proportion of a herd exported from state j. We then randomly sample cows to be exported from the 4 epidemiological compartments of the origin herd, where $S_{i,j}^{\text{exported}}$ is the number of cows that will be removed (exported) from the susceptible compartment of herd i in state j, and likewise for the exposed, infected, and recovered compartments:

$$S_{i,j}^{\text{exported}} \sim \text{Binomial}\left(S_{i,j}, P_j^{\text{export size}}\right), \tag{13}$$

$$E_{i,j}^{\text{exported}} \sim \text{Binomial}\left(E_{i,j}, P_j^{\text{export size}})\right), \tag{14}$$

$$I_{i,j}^{\text{exported}} \sim \text{Binomial}\left(I_{i,j}, P_j^{\text{export size}})\right). \tag{15}$$

$$R_{i,j}^{\text{exported}} \sim \text{Binomial}\left(R_{i,j}, P_j^{\text{export size}})\right). \tag{16}$$

If $N_{i,j}^{\text{exported}} = 0$, i.e. no cattle are drawn to be exported, then we repeat the draws of cattle exported, to ensure that there is always at least one cow exported from a herd that has already been probabilistically defined as exporting cattle that time step. We list the values of $P_j^{\text{export size}}$ in Table S4, and plot them in Figure S4 below:

Table S4: The mean proportion of a herd's population that is exported by state.

State	Mean proportion of origin herd's total population that is exported.
Alabama	0.043641
Arizona	0.043284
Arkansas	0.049256
California	0.042684
Colorado	0.043804
Connecticut	0.044499
Delaware	0.047330
Florida	0.041638
Georgia	0.044375
Idaho	0.043360
Illinois	0.041901
Indiana	0.049943
Iowa	0.046084
Kansas	0.046716
Kentucky	0.049534
Louisiana	0.036121
Maine	0.039004
Maryland	0.043558
Massachusetts	0.046212
Michigan	0.043190
Minnesota	0.045885
Mississippi	0.041946
Missouri	0.052611
Montana	0.048579
Nebraska	0.036096
Nevada	0.034981
New Hampshire	0.046465
New Jersey	0.044953
New Mexico	0.040021
New York	0.045065
North Carolina	0.048999
North Dakota	0.047075
Ohio	0.046338
Oklahoma	0.052091
Oregon	0.043457
Pennsylvania	0.046188
Rhode Island	0.038215
South Carolina	0.045295
South Dakota	0.049772
Tennessee	0.049928
Texas	0.042814
Utah	0.046098
Vermont	0.053861
Virginia	0.048895
Washington	0.043427
West Virginia	0.063624
Wisconsin	0.045235
Wyoming	0.060808

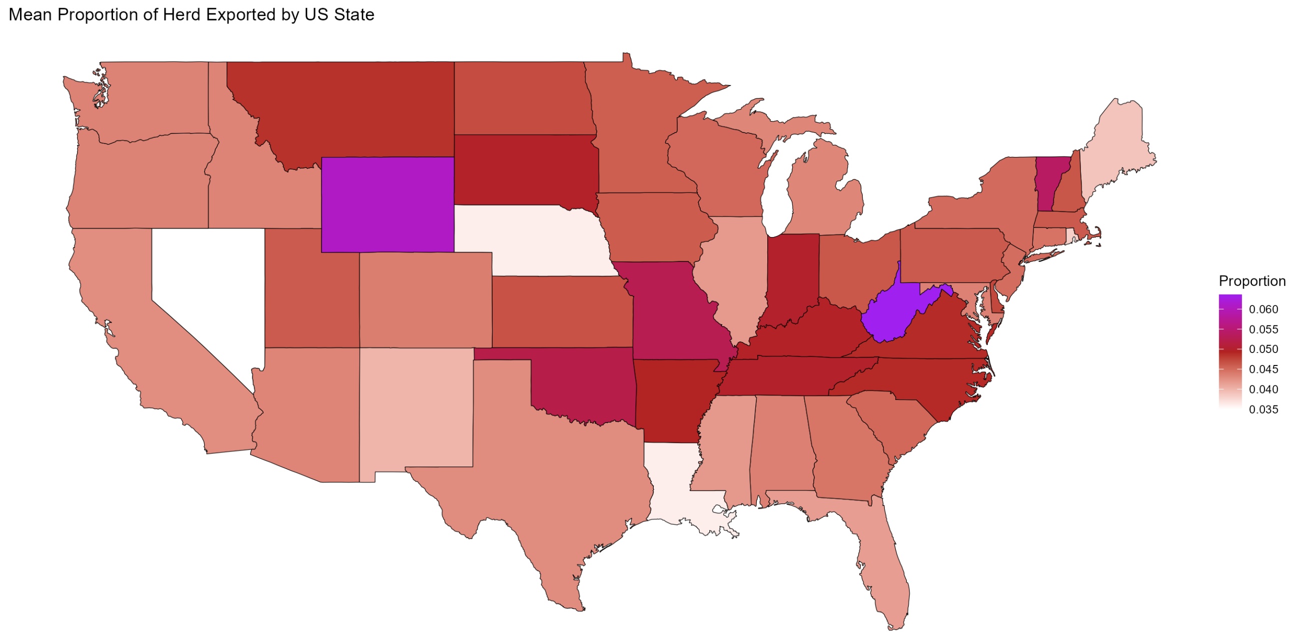

Figure S4: A heatmap of $P_k^{\text{export size}}$, the proportion of a herd that is exported, should an export occur, as given in Table S4. Greater values indicate states which export greater proportions of their herds in each shipment.

We then stochastically decide which herd, and in which state, the exported cattle will be delivered to. The USAMM simulated annual exports data allows us to identify the most common inter-state trading partners for each state. We construct a 48 × 48 movement matrix M, where element $M_{i,j}$ is the probability that cattle exported from state i, will be exported to state j. The rows of matrix M thus all sum to 1. To calculate each value $M_{i,j}$, we tally the total number of exports from state i to state j across all realisations and dates from the USAMM model, and divide by the total number of exports from state i. We present these values in the additional "Supplementary Material 2 - US State Movement Matrix" csv file, however they are also available within our `cowflu` package via `cowflu::movement$movement_matrix`. We plot a heatmap of the movement matrix in Figure S5. As expected, these values mostly align with how geographically close states are, and the size of the destination state's respective dairy industry. We also see that the highest probability of destination is usually (but not always) for the export to go to a different herd within the same state.

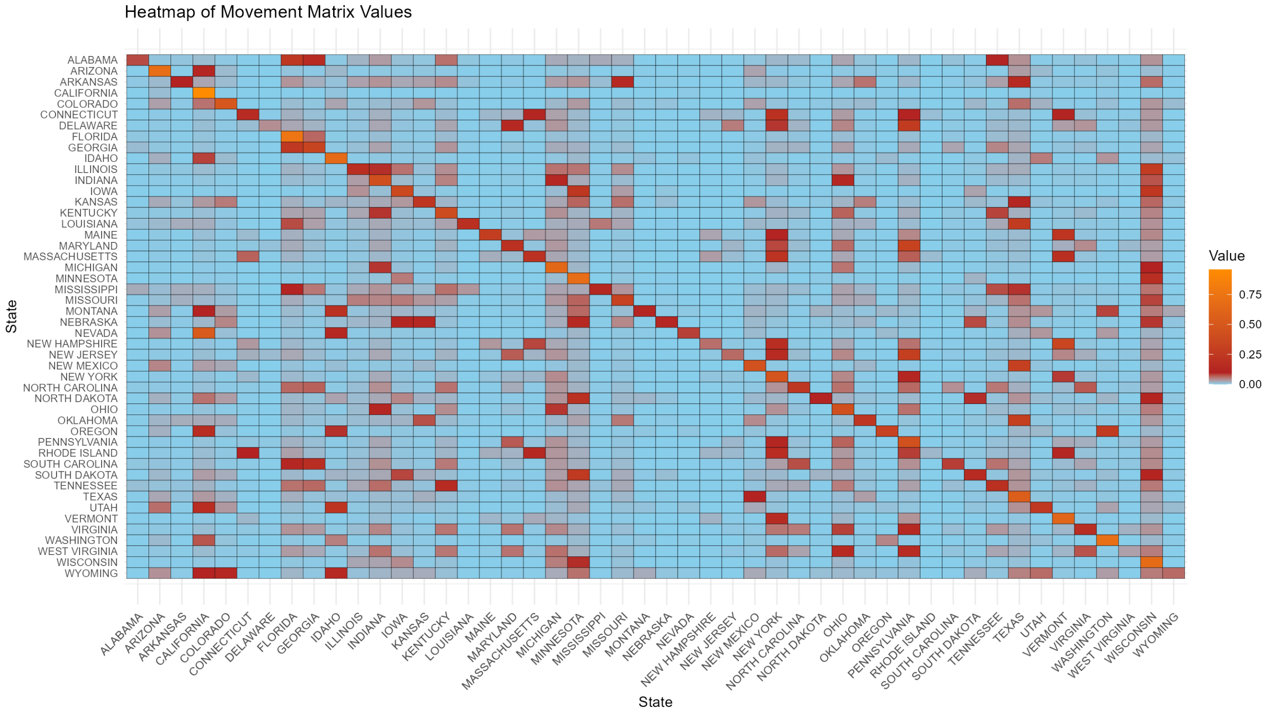

Figure S5: A heatmap of movement matrix M. Should an export from a state i occur, the destination state is randomly determined from row i of the matrix. Hence value $M_{i,j}$ depicts the probability an export from state i will go to state j. The values of each row sum to 1. The diagonal represents the probability that the export of cattle will go to a different herd within the same state.

After stochastically allocating a destination state in accordance with the above movement matrix, we then also stochastically assign which herd in the destination state the exported cattle will be sent to. If a herd was picked at random with equal weighting, this would cause all herd populations to eventually converge to the size herd size, since the number of cattle exported is a proportion of the origin herd's total population. Therefore, this probability is weighted by the respective candidate destination herd's total population. For herd i in the destination state, the probability of the cattle being sent to that respective herd is $\frac{N_i}{\sum_{k=1}^{N_{\text{herds}}} N_k}$.

At this stage, we know i) if the herd in question is exporting cattle, ii) how many cattle are being exported, and iii) which herd and state it is exporting to. However, before moving the cattle from the epidemiological compartments of the origin herd to the destination herd, we have to decide if the exported cattle pass the interstate testing mandates. As detailed in the main manuscript, as of April 29th 2024, cattle exported interstate have up to 30 cows in the cohort tested for H5N1 influenza [5]. Should the herd test positive, the export cannot proceed, and the origin herd must be quarantined for 30 days before being tested again.

If the destination state is the same as the origin state, i.e. the exported cattle are not crossing state boundaries, then the export proceeds - movements within-state are not tested. If the model time step is before April 29th 2024, then the export proceeds. If the destination herd is in a different state, and if the model time step is at a point after April 29th 2024, then we simulate the testing of the exported cattle. If there are less then 30 cattle in the exported cohort, then all the cattle will be tested. Therefore, if there are any infected cattle in the exported cohort, i.e. $I_{i,j}^{\text{exported}} > 0$, then the export does not go ahead, and the cattle remain in their origin herd. Note that cattle in the exposed category, $E_{i,j}^{\text{exported}}$, will still be exported, allowing for a scenario where newly infected cattle, who have not yet incubated the virus long enough to test

positive, will be exported.

If there are more than 30 cattle being exported, we simulate the process of testing 30 cows for infection via a hypergeometric distribution:

$$X \sim \text{Hypergeometric}\left(I_{i,j}^{\text{exported}}, S_{i,j}^{\text{exported}} + E_{i,j}^{\text{exported}} + R_{i,j}^{\text{exported}}, 30\right). \tag{17}$$

i.e. we make 30 draws, without replacement, from a population made up of $I_{i,j}^{\text{exported}}$ "successes", and $S_{i,j}^{\text{exported}} + E_{i,j}^{\text{exported}} + R_{i,j}^{\text{exported}}$ "failures". Thus, if $X > 0$, then an infected cow has been detected in the cohort, and the export does not proceed. If, instead, $X = 0$, then the export of cattle proceeds.

2.5 Epidemiological Parameter Fitting

We fit the five epidemiological parameters detailed in Table S2 via Bayesian evidence synthesis. We build a likelihood function comparing the fit of the step function of "Time of first outbreak detection" for each state to the model simulation of said function. The data extracted from the USDA outbreak portal provides $Y_{j,t}$ - the number of new reported outbreaks in week t in state j. We convert this to the step function, $Y_{j,t}^{\text{first outbreak}}$ like so:

$$Y_{j,\tau}^{\text{first outbreak}} = \begin{cases} 0 & \text{if } Y_{j,t} = 0 \quad \forall\, t < \tau \\ 1 & \text{if } Y_{j,t} \neq 0 \quad \forall\, t < \tau. \end{cases} \tag{18}$$

We use the following likelihood function within our Bayesian evidence synthesis approach;

$$Y_{j,\tau}^{\text{first outbreak}} \sim \text{Bernoulli}(\hat{Y}_{j,\tau}^{\text{first outbreak}}). \tag{19}$$

Where $\hat{Y}_{j,\tau}^{\text{first outbreak}}$, is the model simulation of the "time of first outbreak" function. Therefore,

$$\text{Log-likelihood} = \sum_{j=1}^{48}\sum_{t=1}^{T} Y_{j,t}^{\text{first outbreak}} \log\left(\varepsilon + \hat{Y}_{j,t}^{\text{first outbreak}}\right) + \left(1 - Y_{j,t}^{\text{first outbreak}}\right)\log\left(1 - \varepsilon + \hat{Y}_{j,t}^{\text{first outbreak}}\right), \tag{20}$$

where $\varepsilon = 1\text{e}^{-6}$ is a very small value to avoid the evaluation of log(0).

We also produce all model results using a different likelihood choice - fitting to the number of new weekly reported outbreaks by state, instead of "time of first outbreak detection". This format and results are presented in Section 3.2.1 below.

The fitting is performed via particle Markov Chain Monte Carlo simulation (pMCMC) [6, 7] via the `dust2` and `monty` packages. 16 chains are run for 40,000 iterations. To avoid stuck chains and improve parameter space exploration, particle weighting is reset every 200 iterations. Convergence is assessed via the potential scale reduction factor (psrf), with all parameters displaying a psrf < 1.1. Prior distribution, posterior distribution, and psrf for each parameter is given in Table S5. Note that we fit $\frac{\beta}{\gamma}$ instead of just β to improve chain mixing due to observed correlation between β and γ.

Parameter	Prior Distribution	Posterior median (95% CrI)	psrf	ESS
$\frac{\beta}{\gamma}$	Uniform(0.05, 3)	1.864 (0.929-2.932)	1.06	450.42
α	Uniform(0, 0.1)	0.063 (0.009-0.098)	1.01	2381.91
σ	Uniform(0.05, 2)	1.050 (0.199-1.956)	1.06	478.34
γ	Uniform(0.05, 2)	1.084 (0.384-1.942)	1.08	536.96
A^{asc}	Beta(1, 1)	0.648 (0.091-0.986)	1.01	776.43

Table S5: Prior distributions, posterior distributions, potential scale reduction factors (psrf), and effective samples sizes (ESS) for each model parameter fit.

Figures S6 to S11 plot the chain trajectories for the first 4 of the 16 chains for each parameter, and the associated log-likelihoods. Figures S12 to S16 show the density plots of the prior and posterior distributions for each parameter.

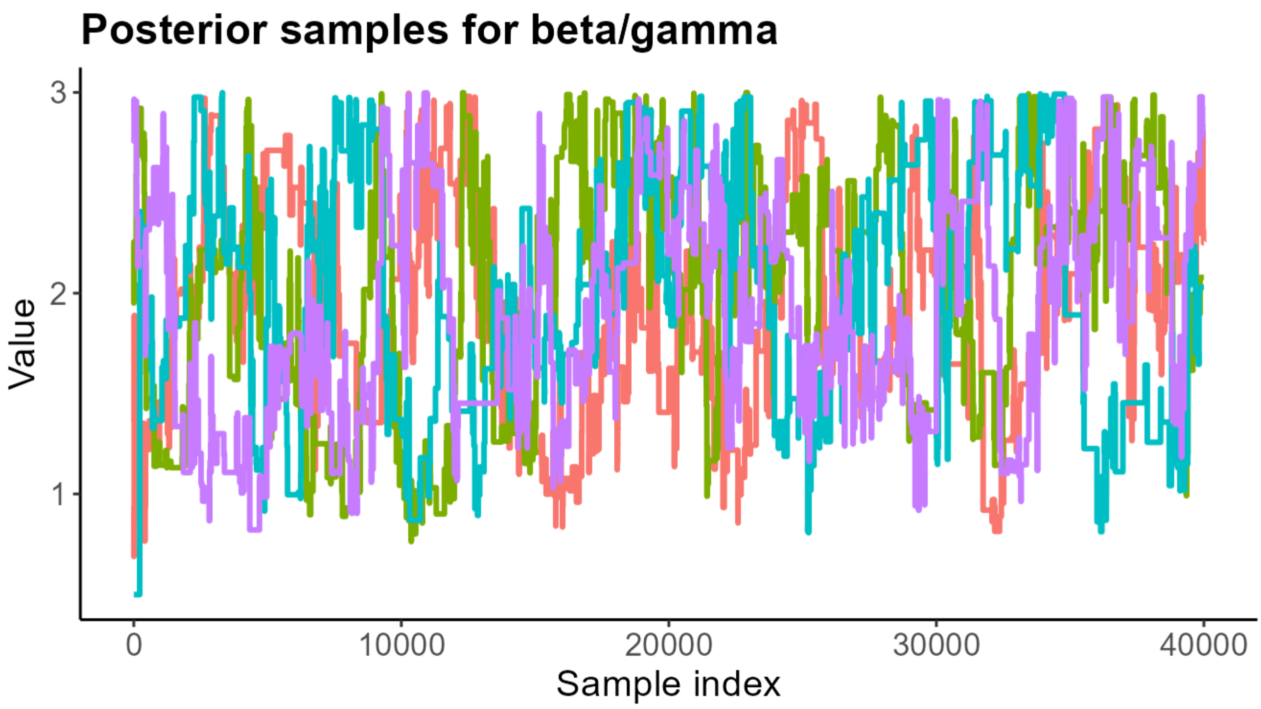

Figure S6: The posterior chain trajectories for all 40000 steps of the pMCMC for the first four of the 16 chains for $\frac{\beta}{\gamma}$.

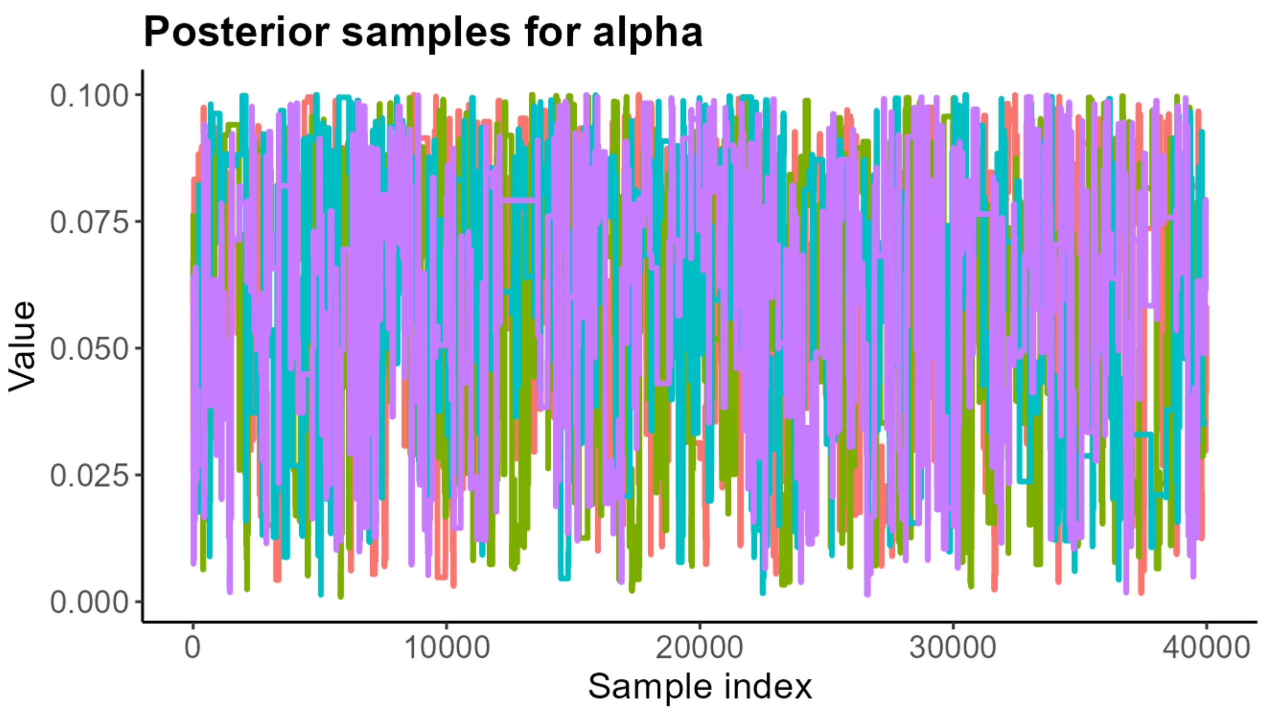

Figure S7: The posterior chain trajectories for all 40000 steps of the pMCMC for the first four of the 16 chains for α.

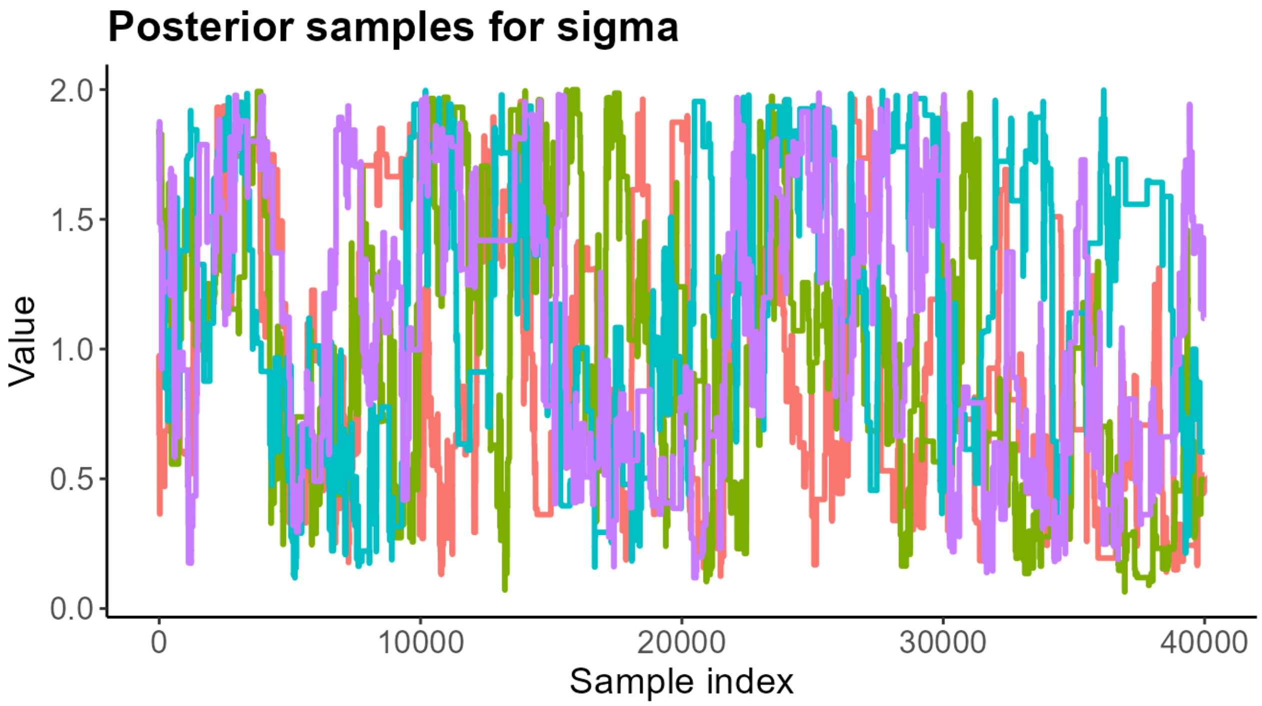

Figure S8: The posterior chain trajectories for all 40000 steps of the pMCMC for the first four of the 16 chains for σ.

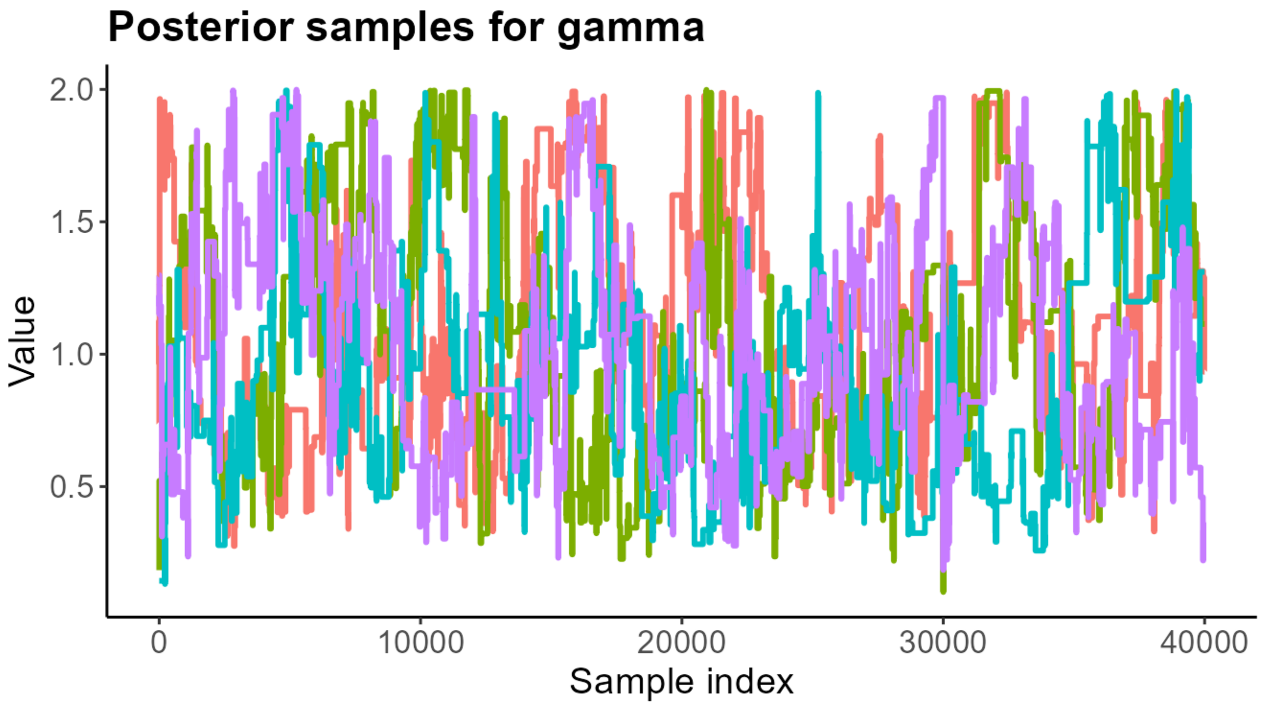

Figure S9: The posterior chain trajectories for all 40000 steps of the pMCMC for the first four of the 16 chains for γ.

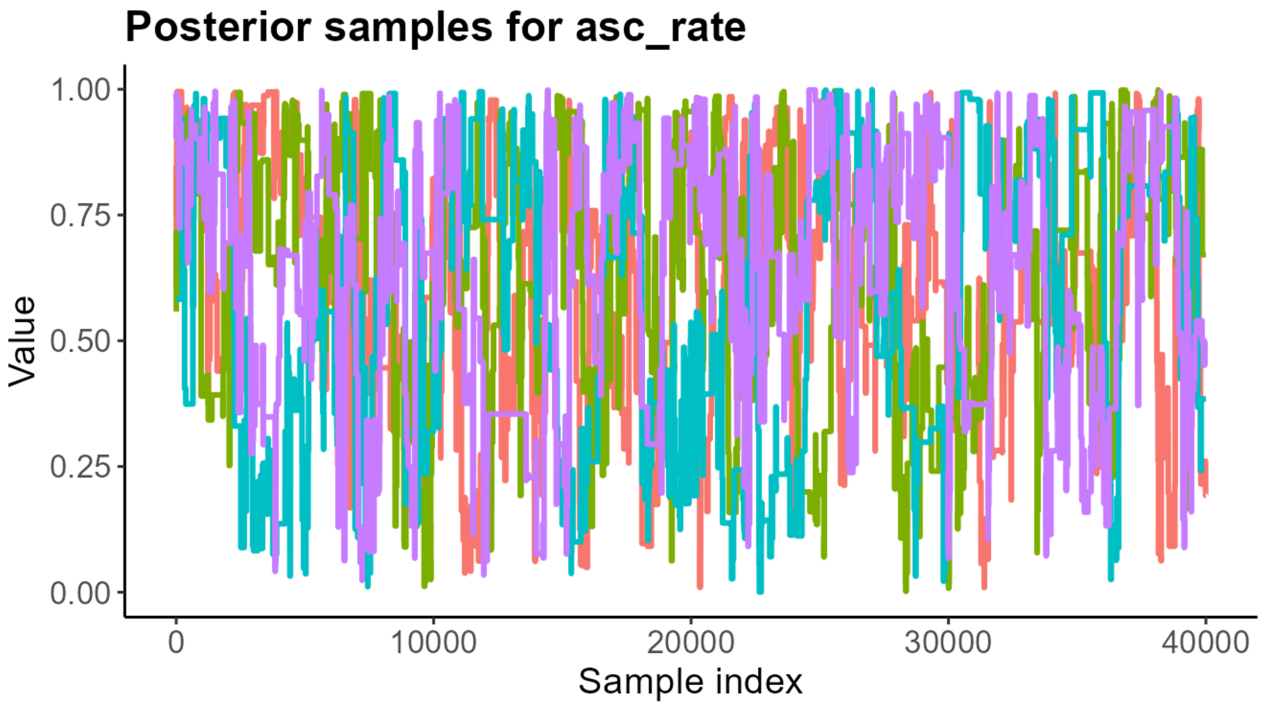

Figure S10: The posterior chain trajectories for all 40000 steps of the pMCMC for the first four of the 16 chains for A^{asc}.

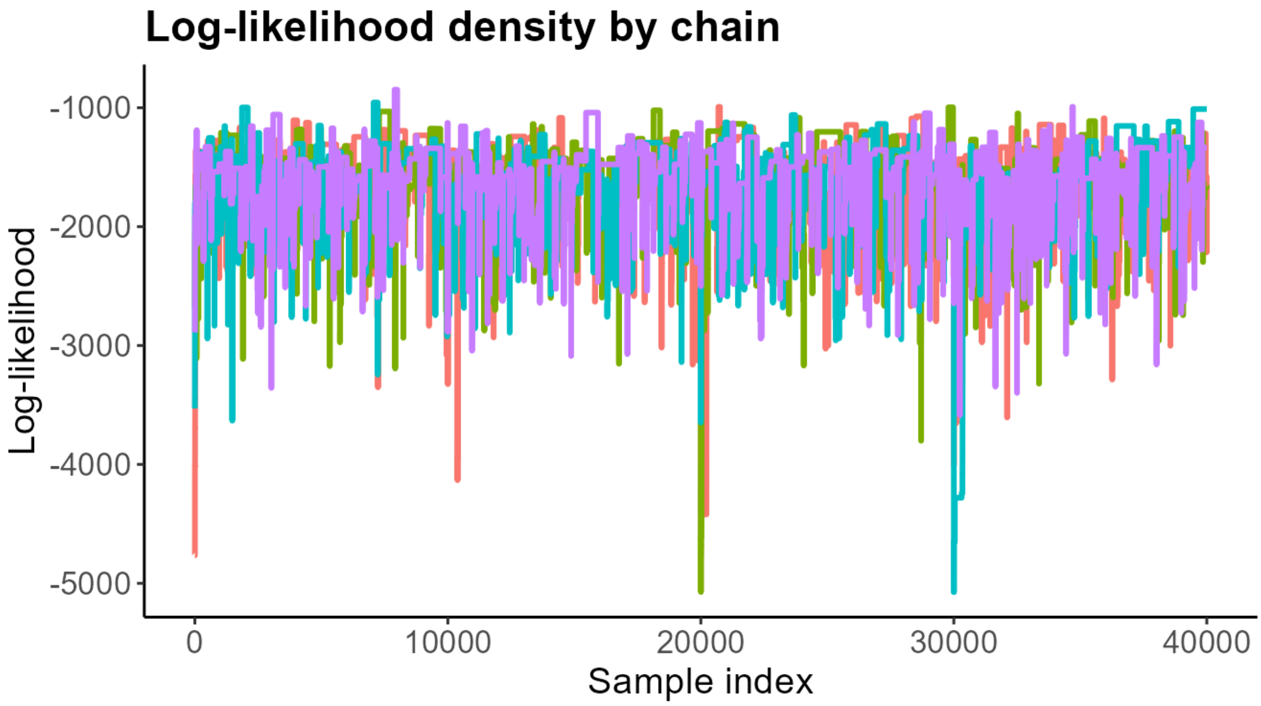

Figure S11: The log-likelihoods for all 40000 steps of the pMCMC for the first four of the 16 chains.

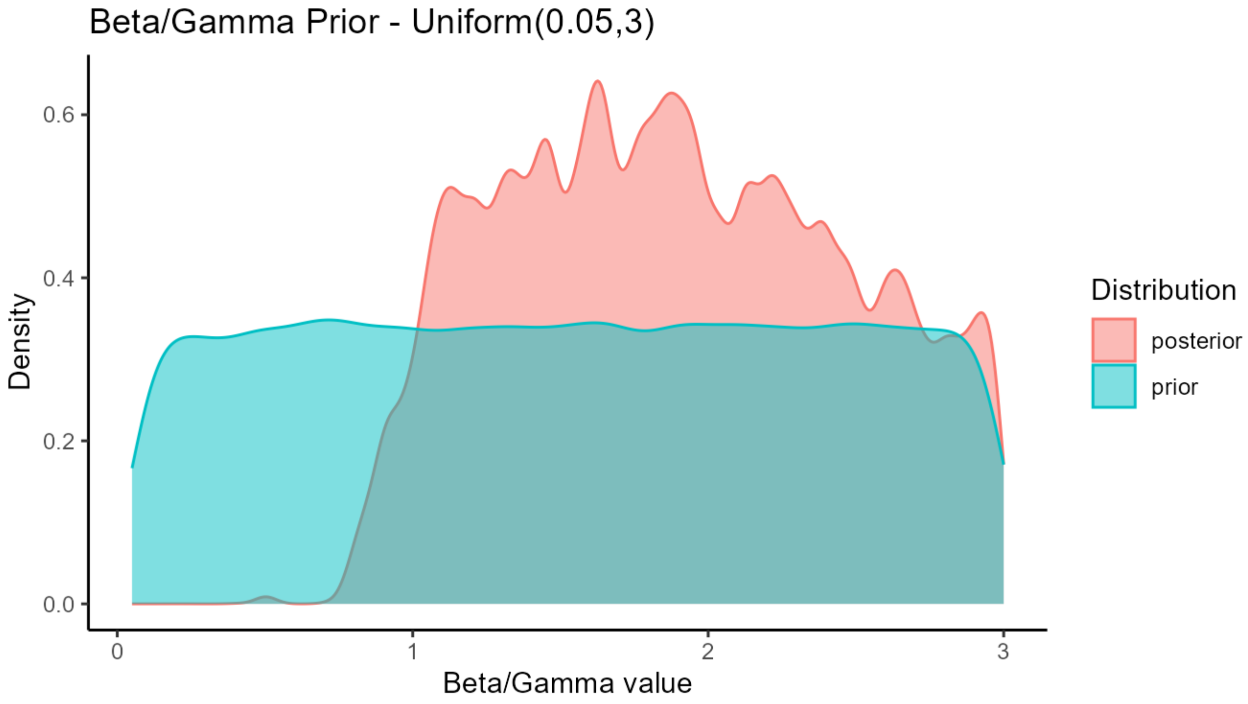

Figure S12: The density plots of the prior and posterior distributions for $\frac{\beta}{\gamma}$.

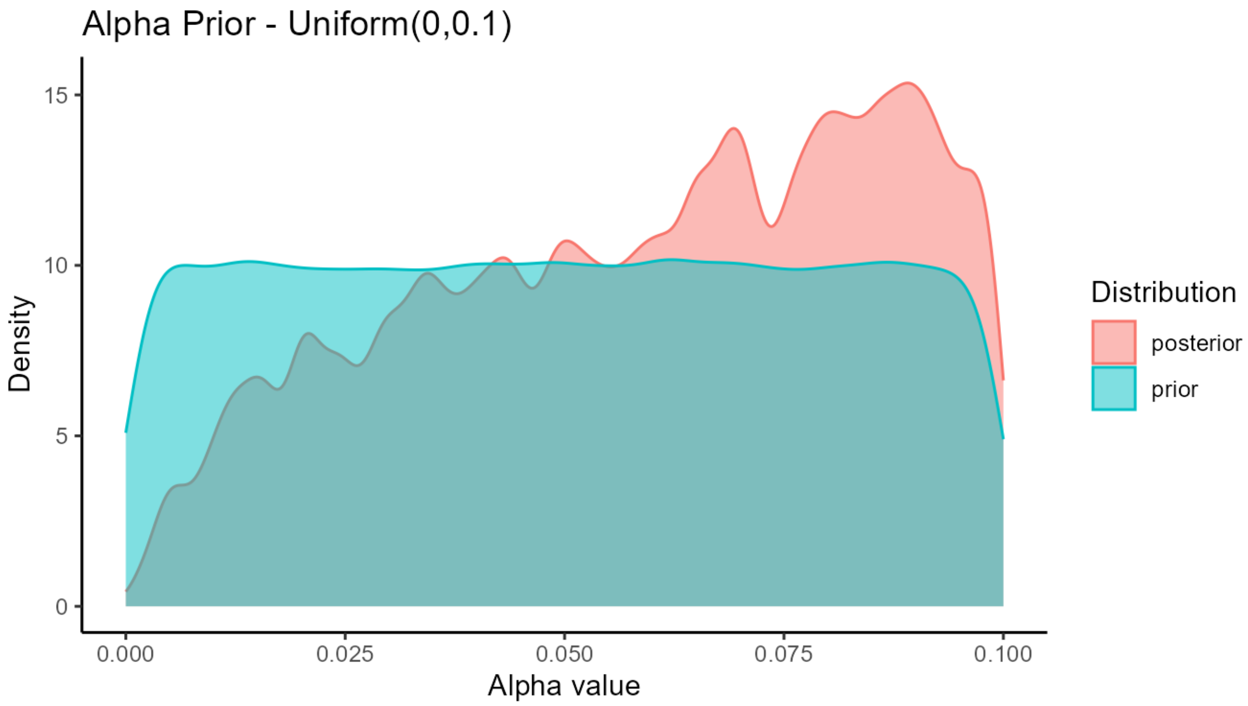

Figure S13: The density plots of the prior and posterior distributions for α.

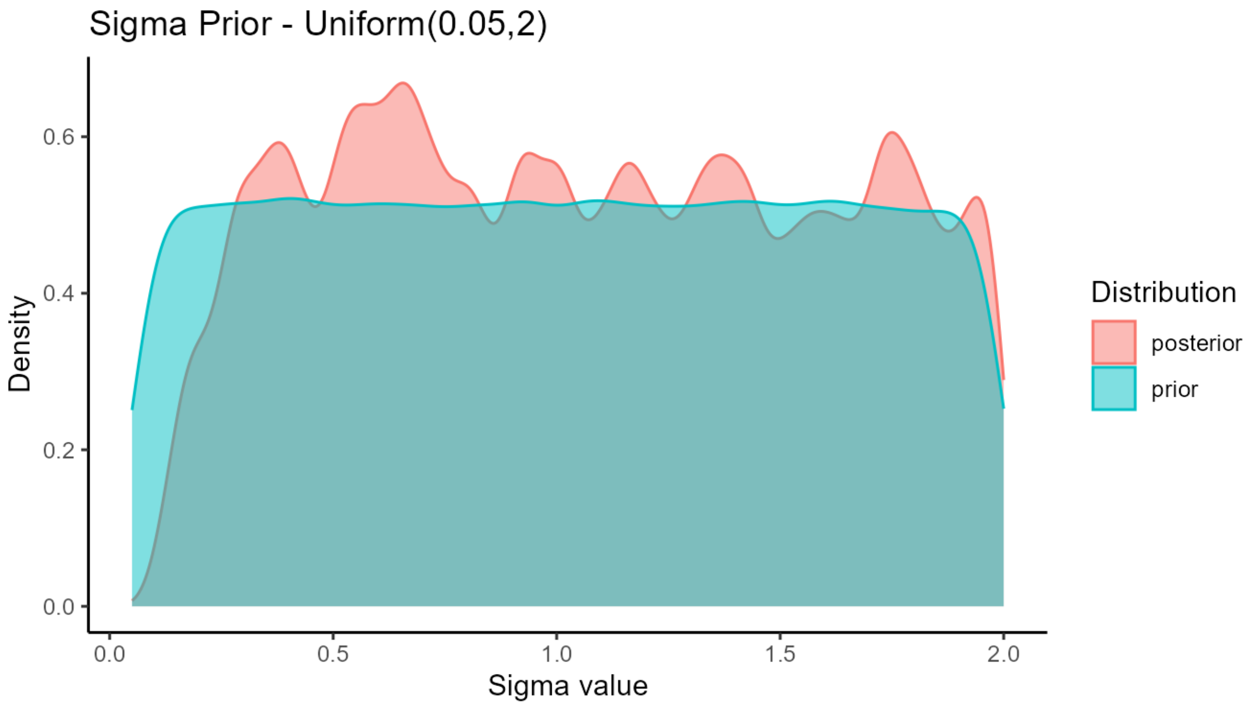

Figure S14: The density plots of the prior and posterior distributions for σ.

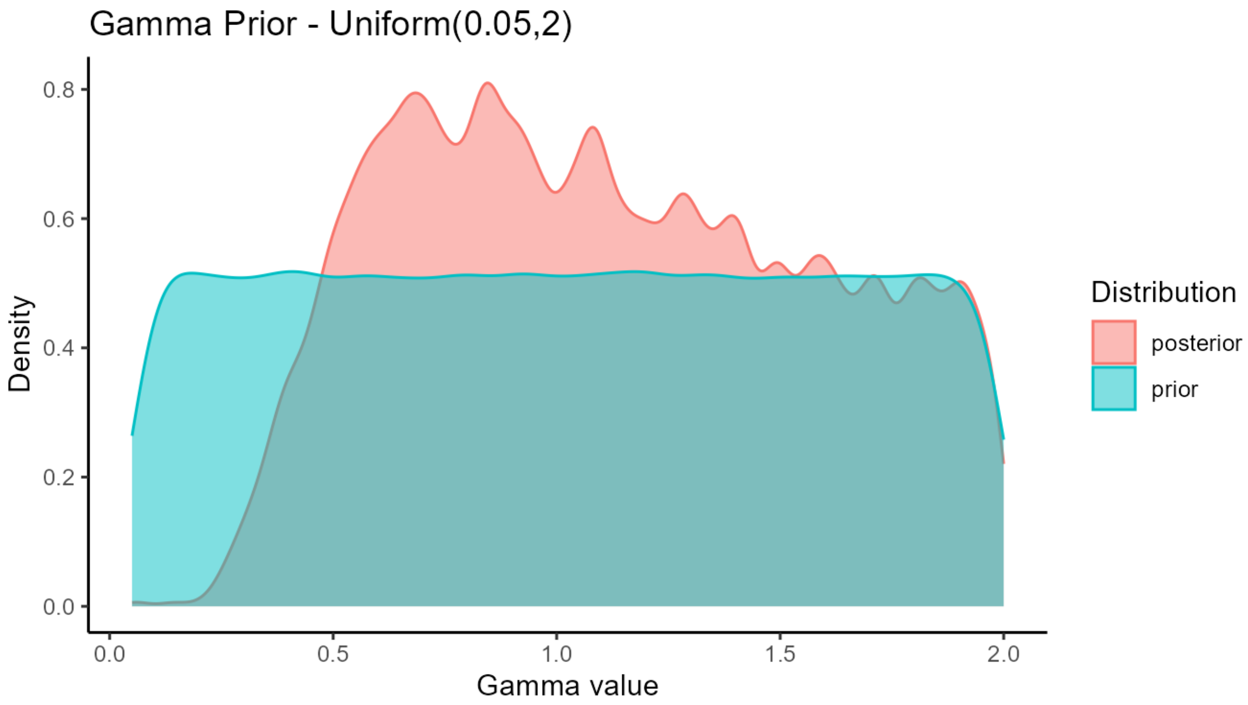

Figure S15: The density plots of the prior and posterior distributions for γ.

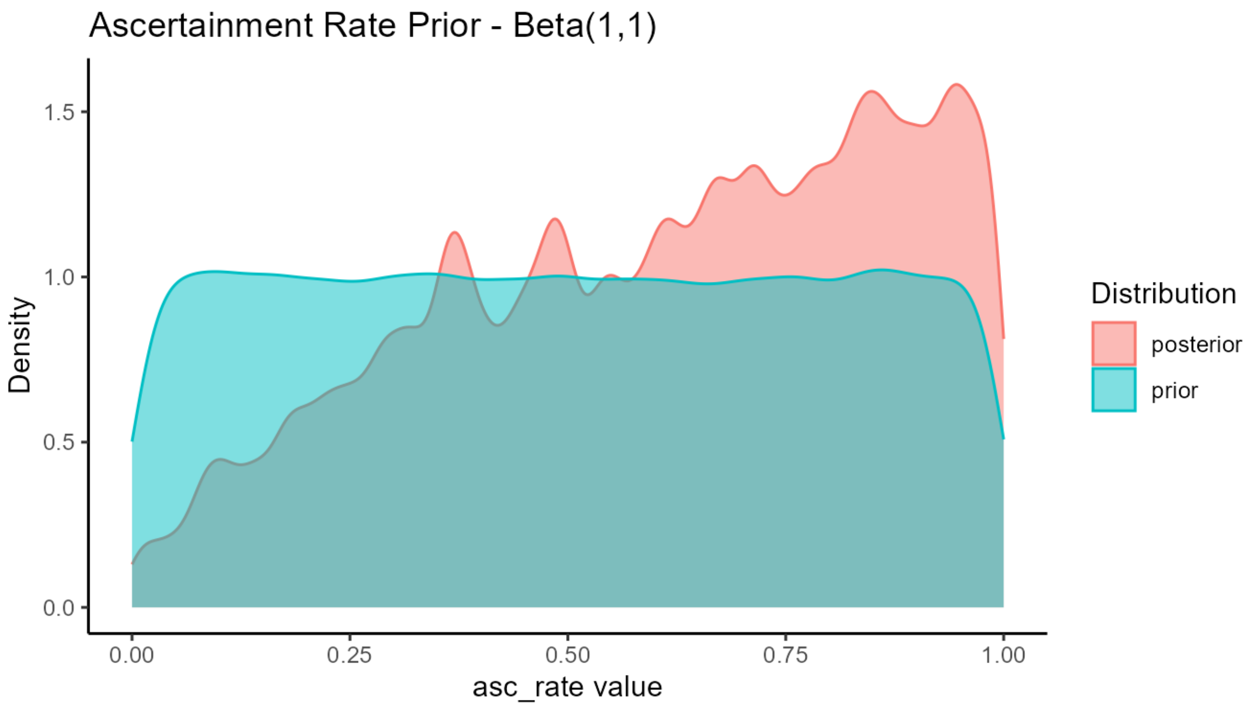

Figure S16: The density plots of the prior and posterior distributions for A^{asc}.

3 Supplementary Results

3.1 Additional Results

Figure 2A in the main manuscript presents the model simulation results for the time of first outbreak reported for each state. Figure 2B presents the model simulation results for the number of new weekly reported outbreaks by state. Figure S17 below plots an additional model output to accompany these results - the "true" proportion of infected herds. i.e. the proportion of herds in each state over time that contain any infected cattle. Figure 2B incorporates the impact of outbreak ascertainment, while Figure S17 does not.

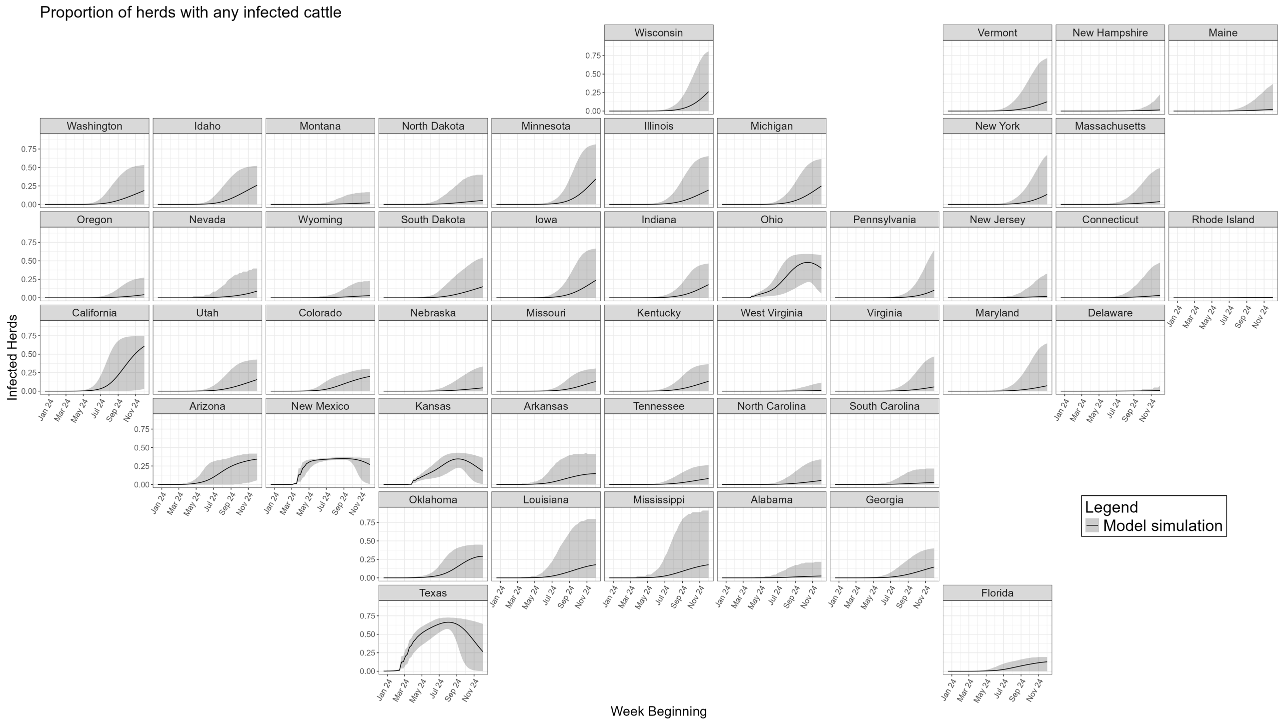

Figure S17: An additional model output accompanying Figure 2 of the main manuscript. The model simulation results of the proportion of herds in each state over time containing any infected cattle. 20,000 model simulations are run, the black line represents the mean result, and the shaded region the 95% CrI.

Figure 4 in the main manuscript displays the mean probability that an exported herd would test positive, for all US states during 3 different weeks over the course of the epidemic. Figure S18 below displays forest plots of those same means but with the associated 95% CrIs.

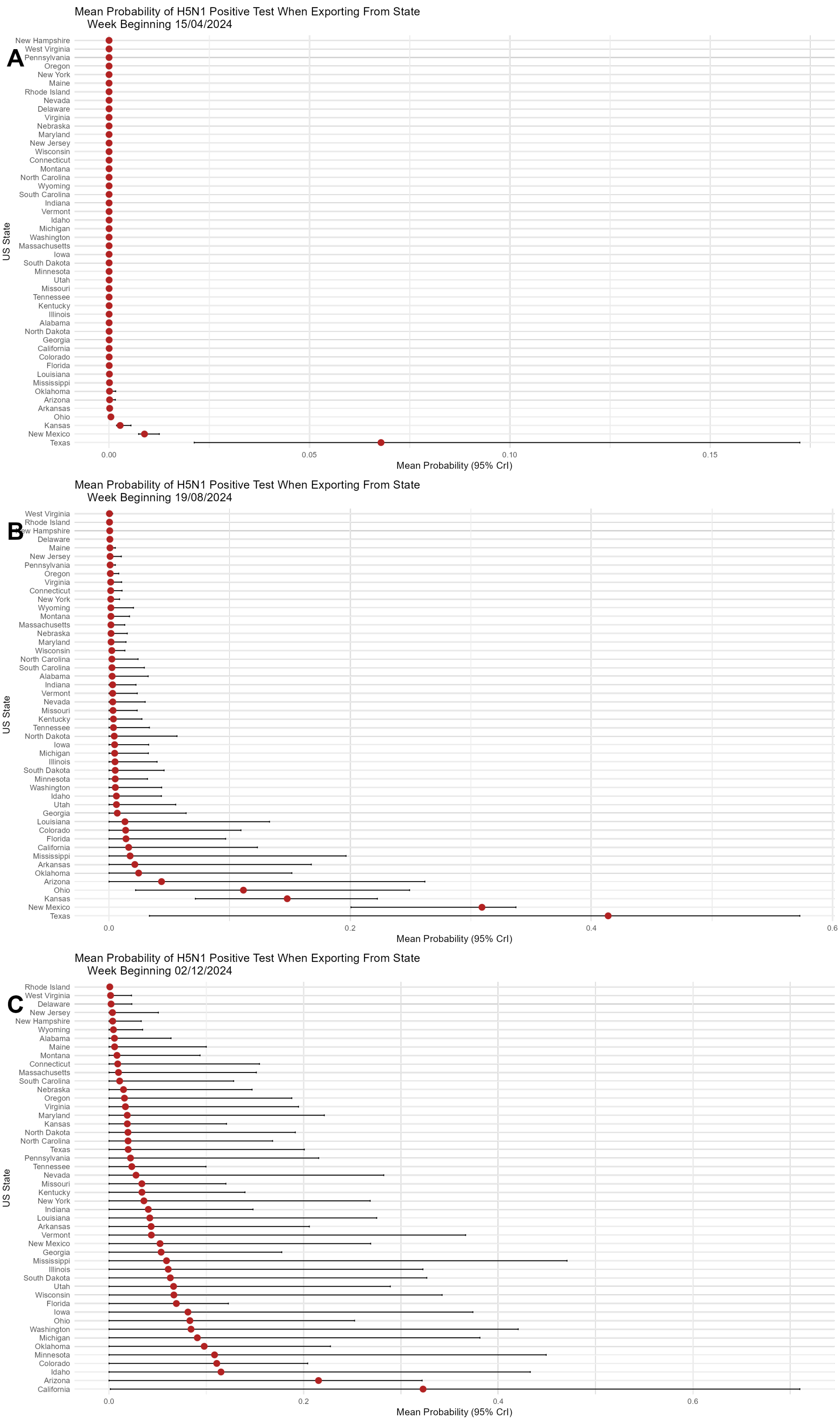

Figure S18: When moving cattle inter-state, up to 30 cattle will be tested for H5N1. Above panels show the state average probability of such an export testing positive over time: **(A)** week beginning April 15th 2024, **(B)** week beginning August 19th 2024, and **(C)** week beginning December 2nd 2024. Red dots indicate mean probability, and black bars the 95% CrIs.

Figure 5 in the main manuscript explores counterfactual scenarios whereby the border-testing intervention is introduced earlier or later, or with a greater number of cows tested. Ultimately, the figure demonstrated that any differences to the intervention had very little impact.

We additionally consider a counterfactual scenario whereby, due to targeted herd-level interventions such as biosecurity improvements through equipment cleaning, workers disinfecting their clothing between sites, or herd quarantining, the rate of spillover between herds can be decreased. Mechanistically, we capture this through changing the α model parameter. The α parameter controls the intra-state transmission of H5N1 between herds in the same US state. It is a fit model parameter. In the baseline analysis, simulations are run by drawing values of α from it's posterior distribution. In the counterfactual scenario, we multiply each draw by 0.5, halving its magnitude. Figure S19 below shows **(A)** the number of new reported outbreaks weekly, **(B)** the number of herds nationally with any infected cattle, and **(C)** the total number of infected cows nationally over time, for both the baseline and counterfactual scenario. The border testing intervention is kept the same in both analyses.

We see that such interventions would have a significant impact on reducing disease burden across all measures, though notably still do not outright stop the spread of disease.

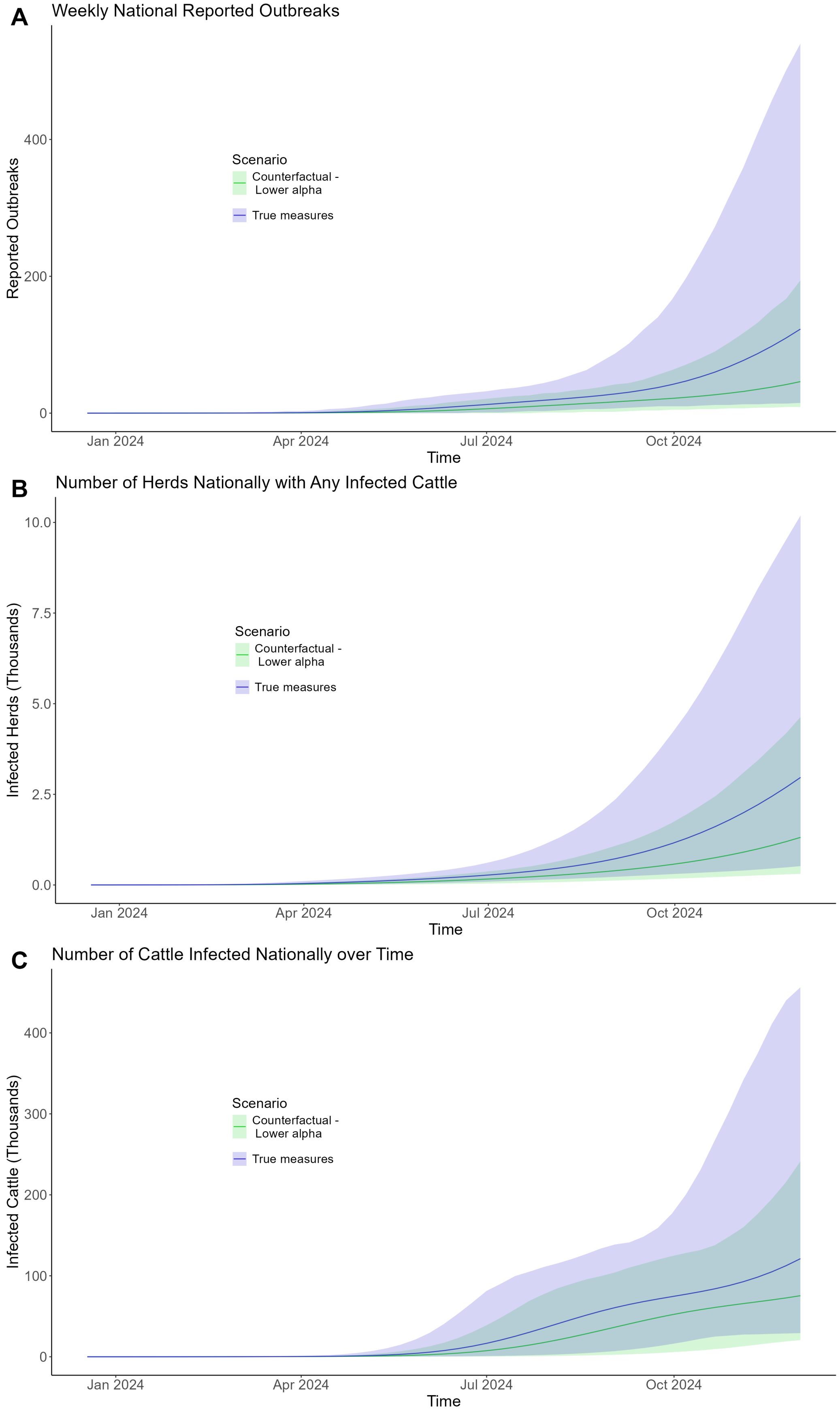

Figure S19: A counterfactual analysis comparing the three model outcomes for both the baseline analysis, and a simulation where the values of α are halved. α represents the intra-state transmission of H5N1 through factors such as shared equipment and staff, or environmental run-off. This counterfactual considers the dynamic impact of improved herd-based biosecurity interventions. **(A)** The number of new reported outbreaks weekly. **(B)** The number of herds nationally with any infected cattle. **(C)** The total number of infected cows nationally over time. Solid lines show simulation mean. Shaded regions show 95% CrI. Blue depicts baseline model assumptions. Green depicts the counterfactual scenario whereby the model parameter α is halved.

To aid interpretation of the significance of herd population, Texas import links, and outbreak detection, we include a visual representation of these combined factors. Figure S20 plots the total number of herds against the mean herd size for each of the 48 continental US states. We see that states with larger mean herd populations appear more likely to have reported a H5N1 outbreak.

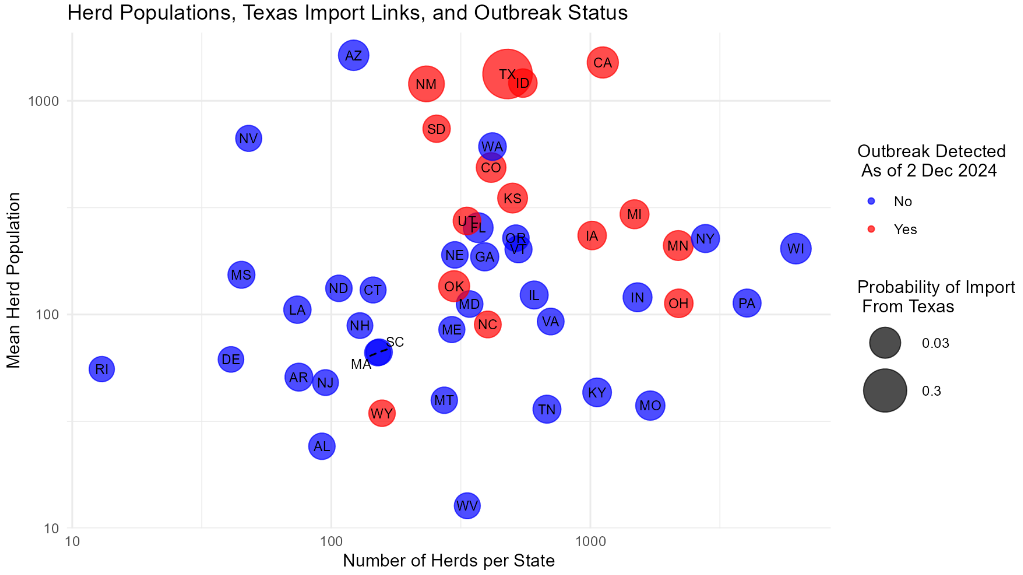

Figure S20: Scatter plot depicting each of the 48 continental US states considered. The x-axis depicts the total number of dairy herds in the state, and the y-axis the mean herd size, calculated as the total number of dairy cattle in the state, divided by the number of herds. Red points depict states which have reported an outbreak of H5N1 in dairy cattle as of December 2nd 2024, and blue points depict those states which have not. The size of the respective points demonstrates the probability that an export of cattle from Texas is sent to that respective state. This means that, the respective values sum to 1, and represent the row of the movement matrix corresponding to Texas.

3.2 Sensitivity Analyses

To explore the implications of modeling decisions, we present alternate versions of all main manuscript figures, but with parameters fit and simulations run under different modeling assumptions. We see that the main findings and presented results are unchanged under these assumptions.

3.2.1 Alternate likelihood

As detailed in Section 2.5 above, our main results are fit using a likelihood function set to the "time of first outbreak detection" instead of weekly outbreak incidence. This is because the number of outbreaks are rarely true independent data samples. When some sites reported outbreaks, some neighbouring farms would be checked or seek testing, meaning the number of reported outbreaks is likely to be extremely auto-correlated. Nonetheless, we also present our results when a likelihood function fitting to the weekly new number of reported outbreaks is used:

$$Y_{j,\tau}^{\text{first outbreak}} \sim \text{Negative Binomial}(\text{mean} = \hat{Y}_{j,\tau}^{\text{first outbreak}}, \text{dispersion} = 1). \tag{21}$$

Figures S21 to S23 below present new versions of Figures 2, 4, and 5 from the main manuscript reproduced under this modeling choice.

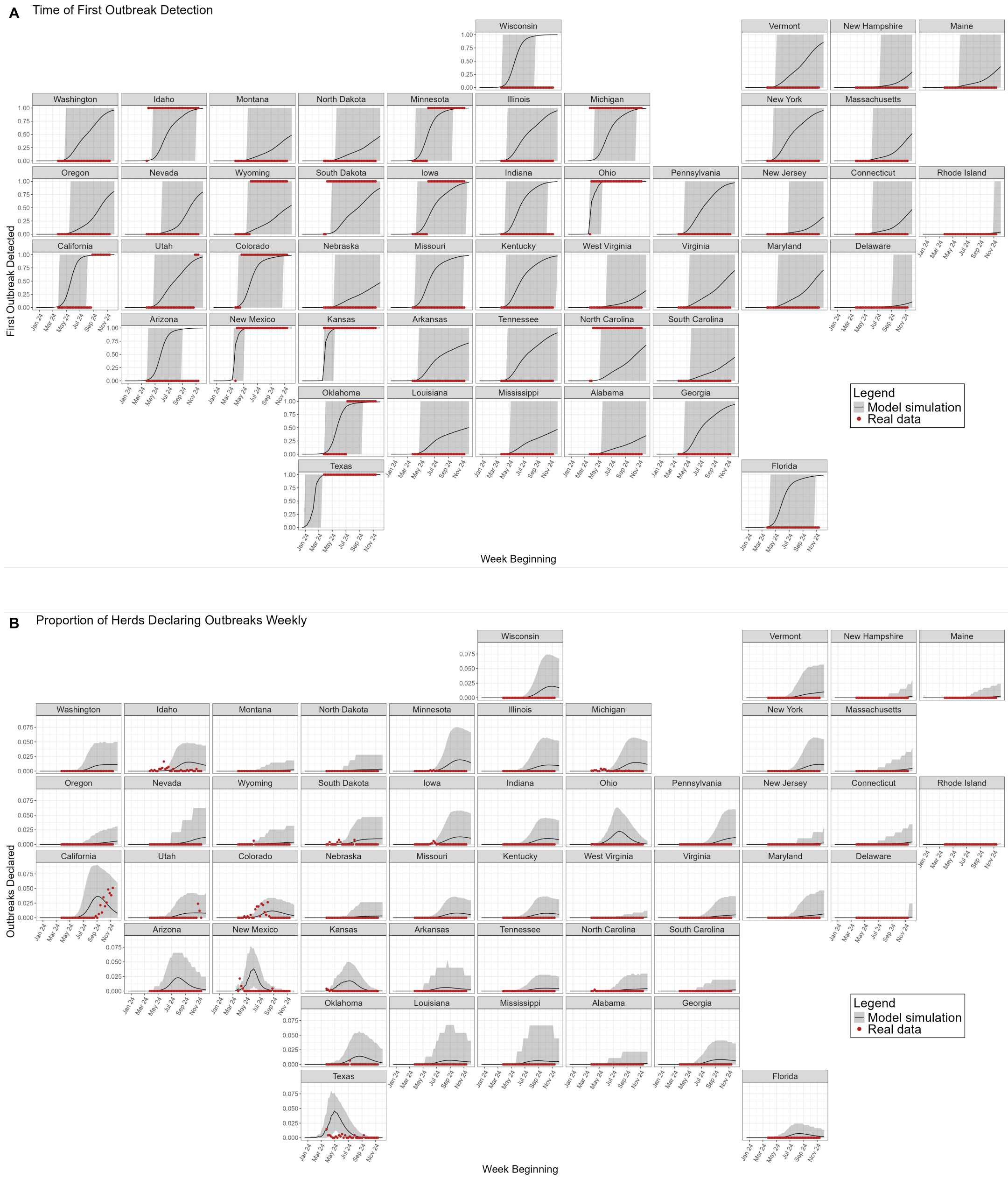

Figure S21: This is a recreation of Figure 2 from the main manuscript when using a likelihood function fitting to new weekly reported outbreaks. **(A)** shows the time at which the first outbreak is detected in a state. **(B)** shows the proportion of herds in each state which report new outbreaks per state each week, accounting for under-reporting. Red points depict real world data. The black line depicts the model mean, the shaded grey region depicts the 95% credible interval (95% CrI).

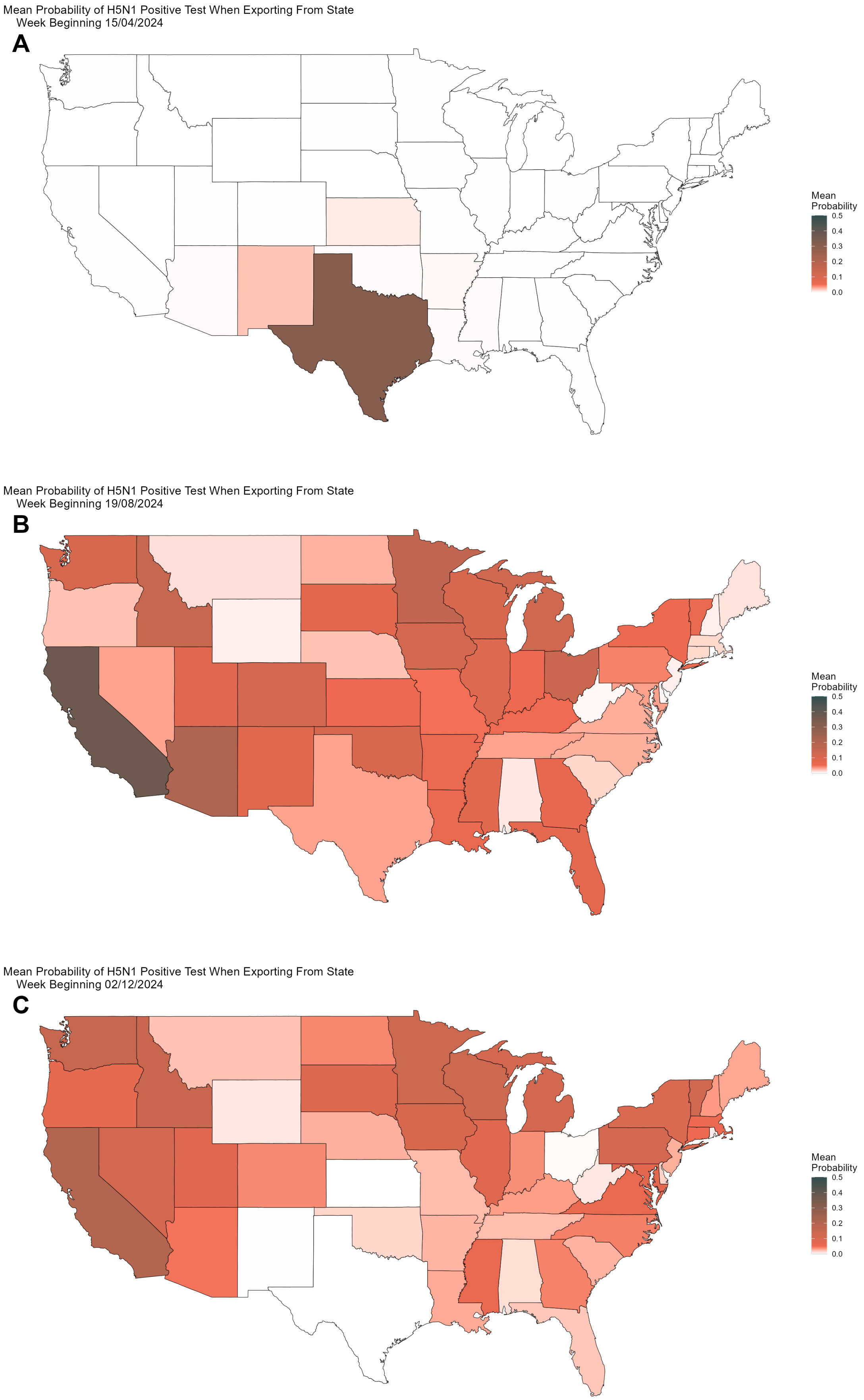

Figure S22: This is a recreation of Figure 4 from the main manuscript when using a likelihood function fitting to new weekly reported outbreaks. When moving cattle inter-state, up to 30 cattle will be tested for H5N1. Above panels show the state average probability of such an export testing positive over time: **(A)** week beginning April 15th 2024, **(B)** week beginning August 19th 2024, and **(C)** week beginning December 2nd 2024.

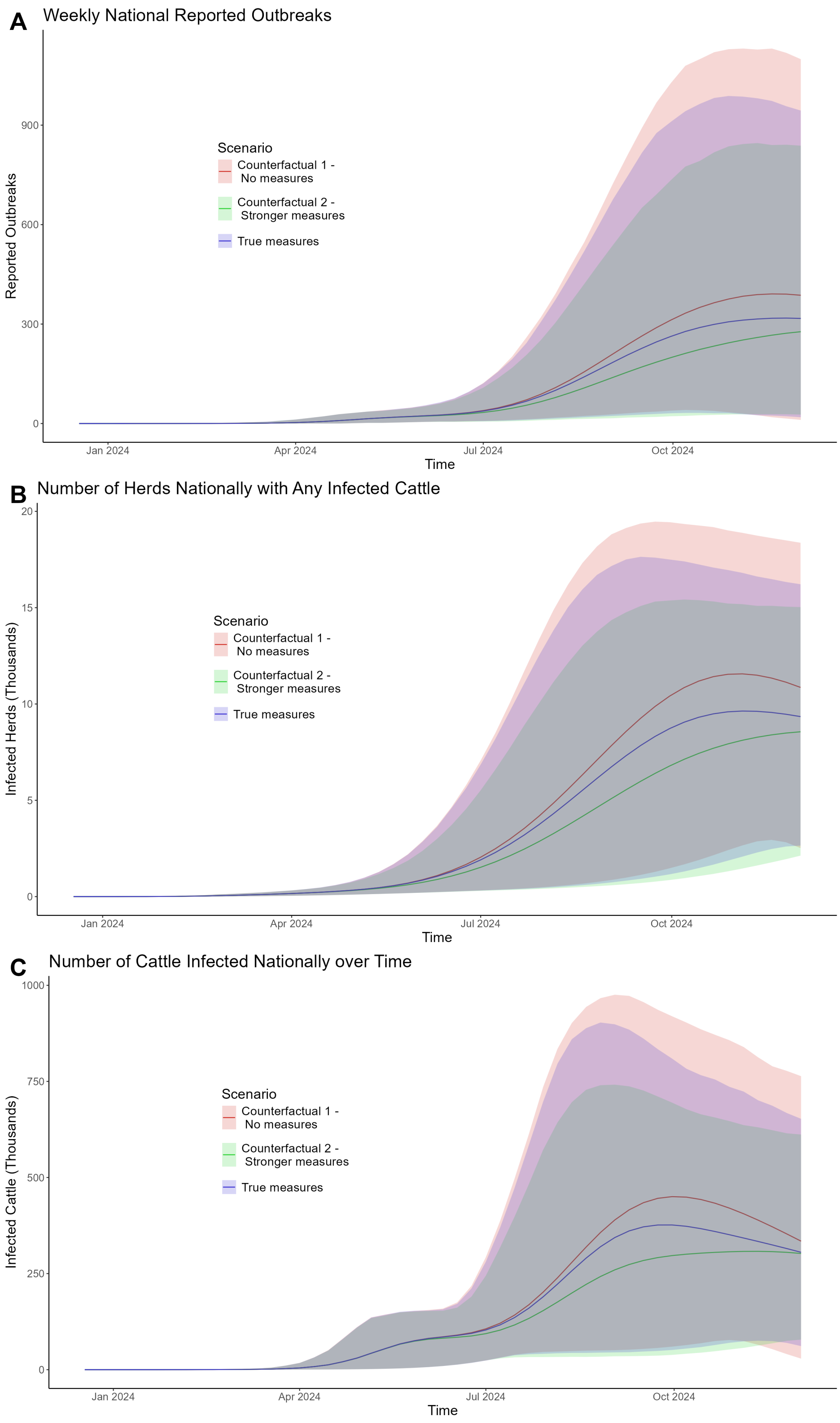

Figure S23: This is a recreation of Figure 5 from the main manuscript when using a likelihood function fitting to new weekly reported outbreaks. **(A)** The number of new reported outbreaks weekly. **(B)** The number of herds nationally with any infected cattle. **(C)** The total number of infected cows nationally over time. Solid lines show simulation mean. Shaded regions show 95% CrI. Blue depicts baseline model assumptions. Red depicts the scenario with no border testing. Green depicts border testing of up to 100 cows, implemented 28 days earlier, on April 1st 2024.

3.2.2 Real ICVI data

All the model assumptions on probabilities of export and movement matrices are based upon USAMM model outputs, as detailed in section 2.4 above. While this is the most comprehensive source of information regarding the inherent uncertainty regarding cattle movements, alternate data streams are available. We were provided the real world ICVI data gathered by Cabezas et al. (2021) [8] detailing a series of exact cattle shipments for the year 2016. Using the same methods as detailed above using the USAMM results, we reproduce our probability of export (Table S3) and movement matrix (**??**) using this data instead.

Figures S24 to S26 below present new versions of Figures 2, 4, and 5 from the main manuscript reproduced with model fits and simulations using these movement probabilities instead.

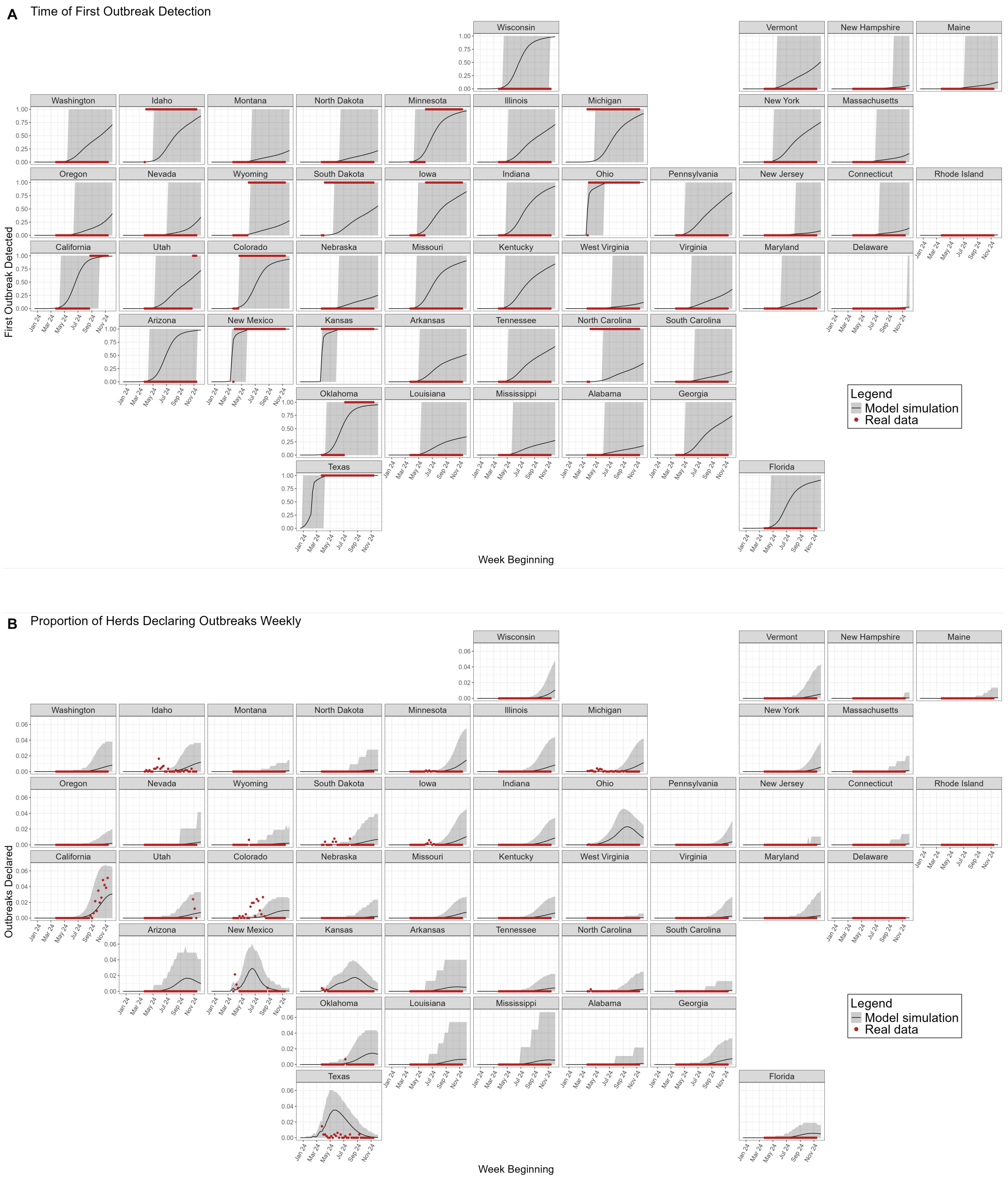

Figure S24: This is a recreation of Figure 2 from the main manuscript when using movement probabilities directly calculated from 2016 ICVI data. **(A)** shows the time at which the first outbreak is detected in a state. **(B)** shows the proportion of herds in each state which report new outbreaks per state each week, accounting for under-reporting. Red points depict real world data. The black line depicts the model mean, the shaded grey region depicts the 95% credible interval (95% CrI).

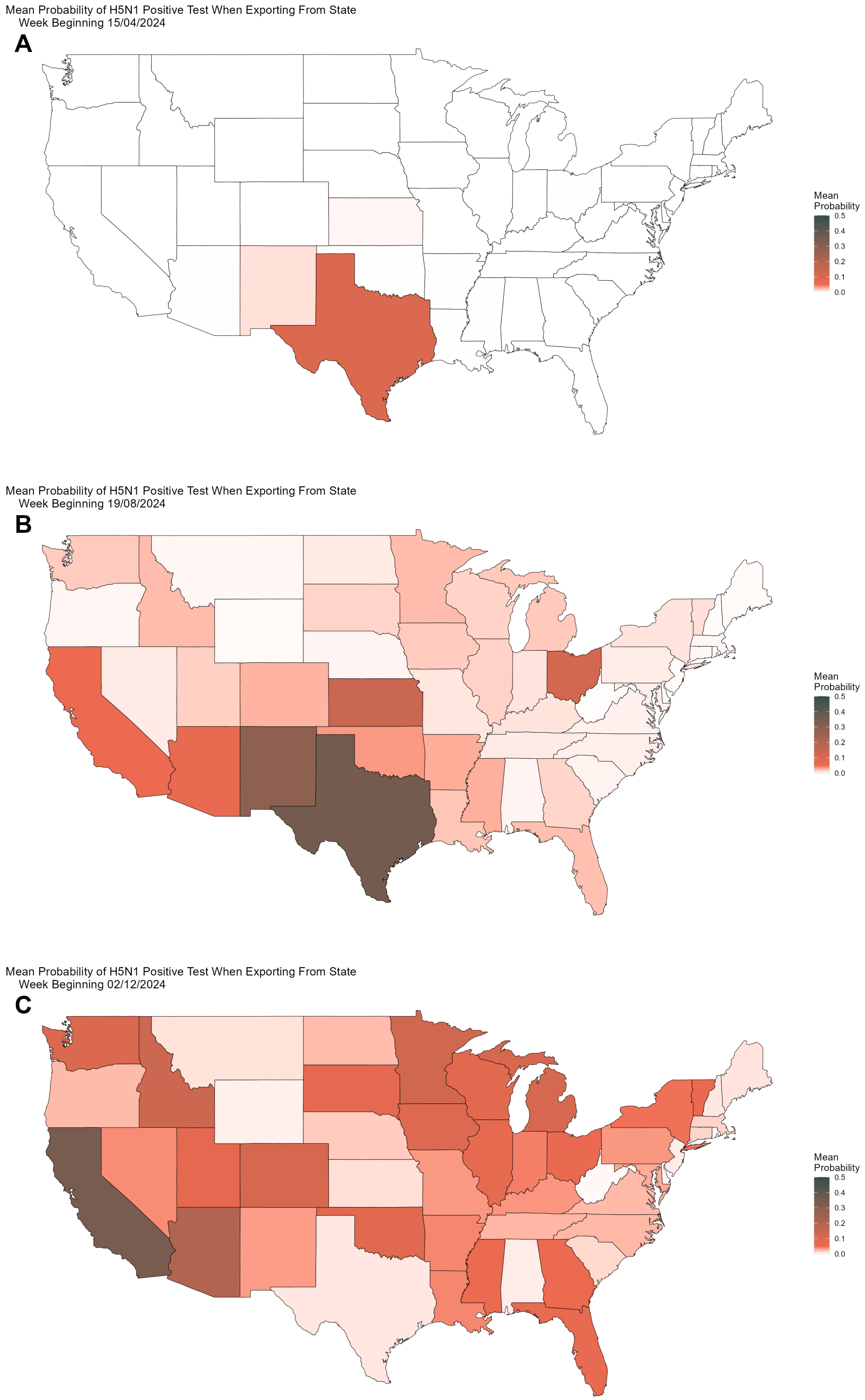

Figure S25: This is a recreation of Figure 4 from the main manuscript when using movement probabilities directly calculated from 2016 ICVI data. When moving cattle inter-state, up to 30 cattle will be tested for H5N1. Above panels show the state average probability of such an export testing positive over time: **(A)** week beginning April 15th 2024, **(B)** week beginning August 19th 2024, and **(C)** week beginning December 2nd 2024.

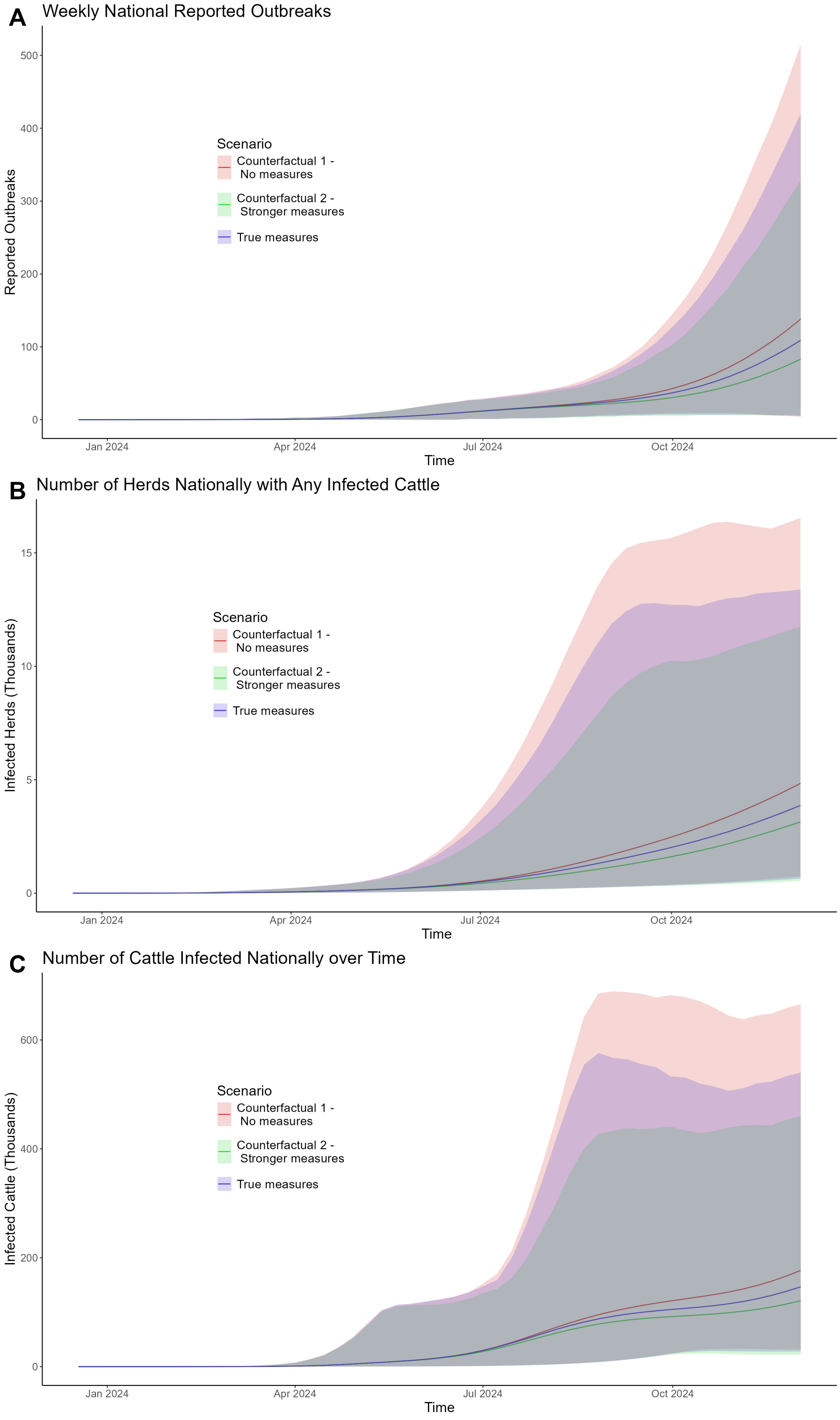

Figure S26: This is a recreation of Figure 5 from the main manuscript when using movement probabilities directly calculated from 2016 ICVI data. **(A)** The number of new reported outbreaks weekly. **(B)** The number of herds nationally with any infected cattle. **(C)** The total number of infected cows nationally over time. Solid lines show simulation mean. Shaded regions show 95% CrI. Blue depicts baseline model assumptions. Red depicts the scenario with no border testing. Green depicts border testing of up to 100 cows, implemented 28 days earlier, on April 1st 2024.

3.2.3 Alternate Ascertainment

The base model assumes that the probability of a herd reporting an outbreak is dependent on the absolute number of infected cattle in the herd, and the proportion of the herd is infected. These assumptions are outlaid in Equation (12) above.

In this sensitivity analysis, we re-fit the model and produce all model results using a more relaxed model assumption, where ascertainment is scaled just by the proportion of the herd that is infected. Specifically, we replace Equation (12) with:

$$\phi_i = \left(\frac{I_i}{N_i}\right) A^{\text{asc}}\, dt. \qquad (22)$$

Figures S27 to S29 below present new versions of Figures 2, 4, and 5 from the main manuscript reproduced with model fits and simulations using these movement probabilities instead.

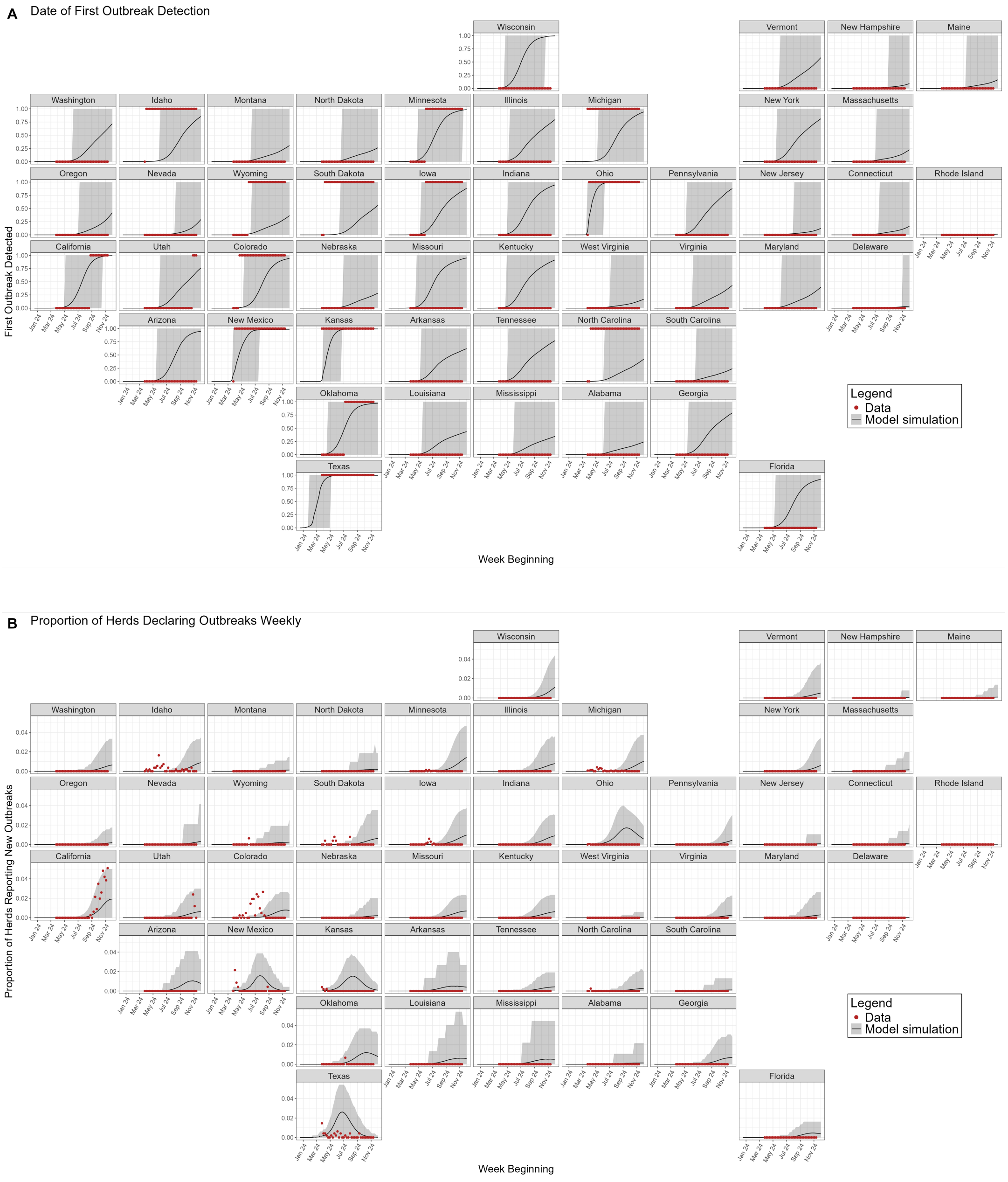

Figure S27: This is a recreation of Figure 2 from the main manuscript when using alternate ascertainment rate assumptions as outlined in Equation (22). **(A)** shows the time at which the first outbreak is detected in a state. **(B)** shows the proportion of herds in each state which report new outbreaks per state each week, accounting for under-reporting. Red points depict real world data. The black line depicts the model mean, the shaded grey region depicts the 95% credible interval (95% CrI).

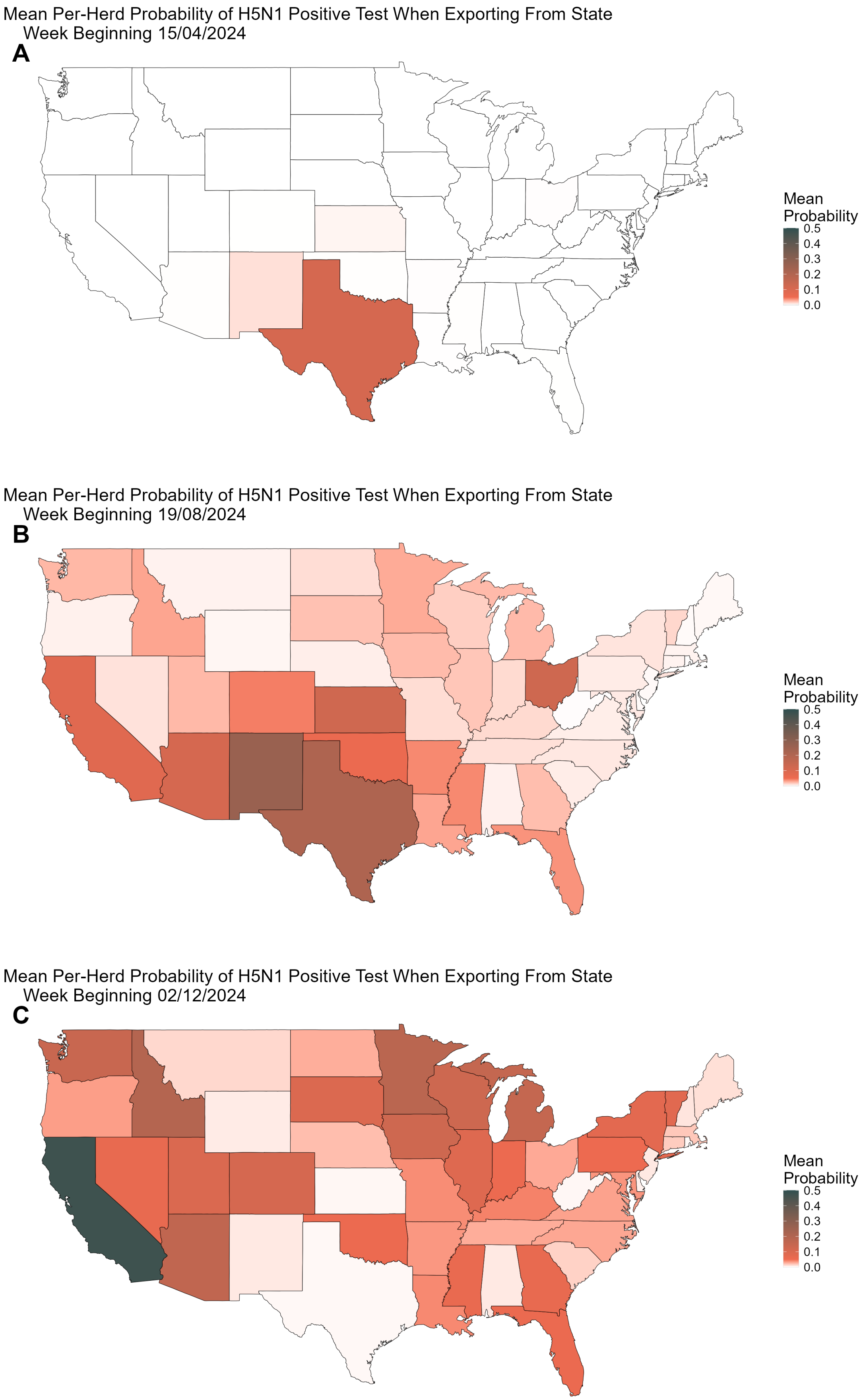

Figure S28: This is a recreation of Figure 4 from the main manuscript when using alternate ascertainment rate assumptions as outlined in Equation (22). When moving cattle inter-state, up to 30 cattle will be tested for H5N1. Above panels show the state average probability of such an export testing positive over time: **(A)** week beginning April 15th 2024, **(B)** week beginning August 19th 2024, and **(C)** week beginning December 2nd 2024.

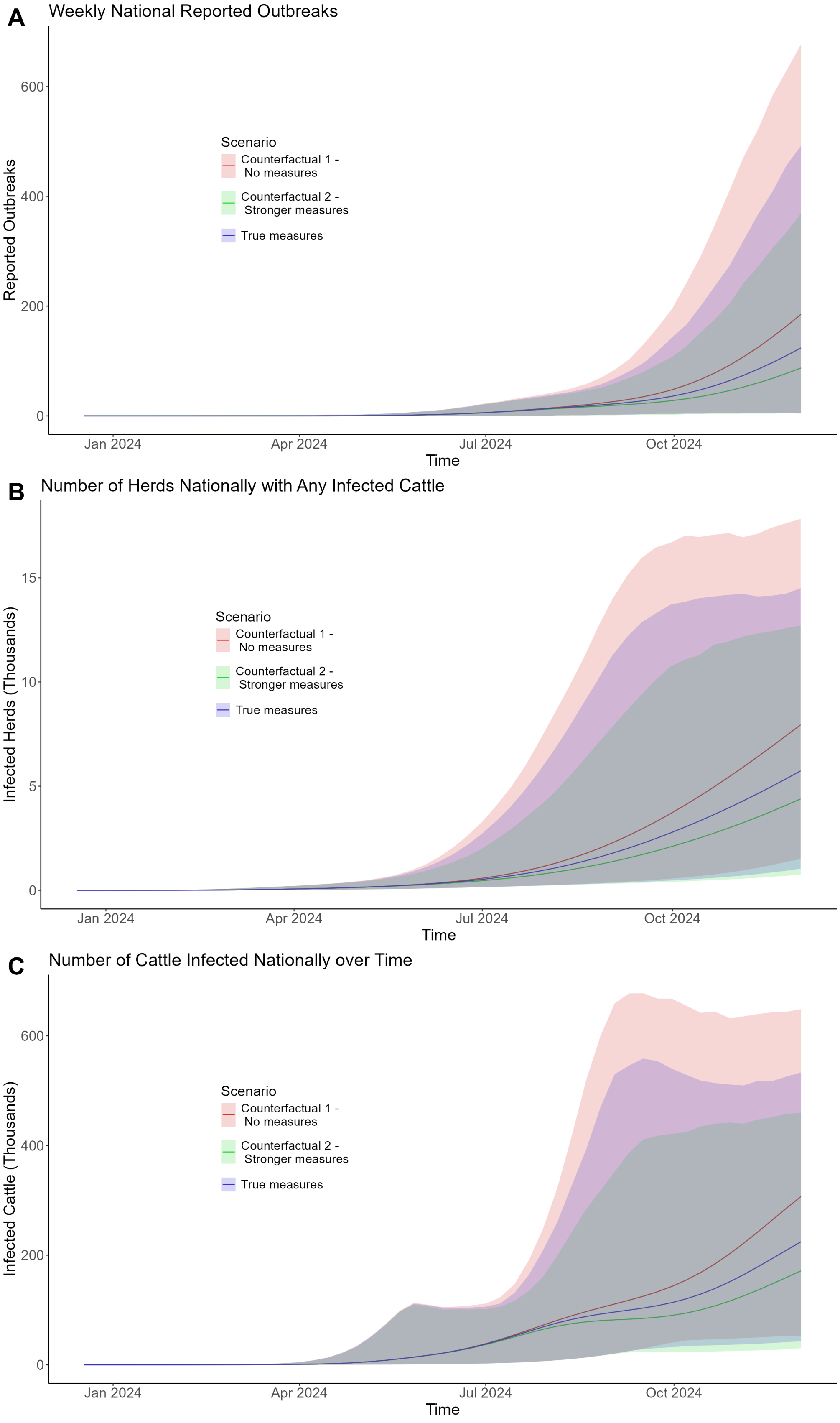

Figure S29: This is a recreation of Figure 5 from the main manuscript when using alternate ascertainment rate assumptions as outlined in Equation (22). **(A)** The number of new reported outbreaks weekly. **(B)** The number of herds nationally with any infected cattle. **(C)** The total number of infected cows nationally over time. Solid lines show simulation mean. Shaded regions show 95% CrI. Blue depicts baseline model assumptions. Red depicts the scenario with no border testing. Green depicts border testing of up to 100 cows, implemented 28 days earlier, on April 1st 2024.

3.2.4 Birth and Death Processes

The base model assumes that the total cattle population remains static. New dairy cows are not born (adding to the population) and nor do dairy cows die, be it through natural mortality, or targeted culling. This decision was made based on the relatively short time frame of the simulation period, but also due to unclear information as to the true rates of disease-related mortality or culling. Early epidemiological reports identified that "Mortality and culling is 2% or less on average" [2], although this would be based only on those outbreaks that were successfully identified.

In this sensitivity analysis, we explore the impact that such birth-death processes have on the underlying model dynamics. To do this, we recreate all figures from the main manuscript, with a model whose underlying equations are adapted from Equations (5) to (8) to:

$$\frac{dS_{i,j}}{dt} = -\beta S_{i,j}\left(\frac{I_{i,j}}{N_{i,j}} + \alpha\frac{I_{-i,j}}{N_{-i,j}}\right) + \mu N_{i,j} - \mu S_{i,j}, \tag{23}$$

$$\frac{dE_{i,j}}{dt} = \beta S_{i,j}\left(\frac{I_{i,j}}{N_{i,j}} + \alpha\frac{I_{-i,j}}{N_{-i,j}}\right) - \sigma E_{i,j} - \mu E_{i,j}, \tag{24}$$

$$\frac{dI_{i,j}}{dt} = \sigma E_{i,j} - \gamma I_{i,j} - \mu I_{i,j}, \tag{25}$$

$$\frac{dR_{i,j}}{dt} = \gamma I_{i,j} - \mu R_{i,j}. \tag{26}$$

The additions, in blue, capture births and deaths subject to the population size of each herd, with the average birth and death rates of equal magnitude, μ. We fix $\mu = \frac{1}{305\,\text{weeks}}$, based on data that the average US dairy cow lifespan is 5.87 years [9].

Figures S30 to S32 below present new versions of Figures 2, 4, and 5 from the main manuscript reproduced with simulations using these additional birth-death processes instead. No significant difference from the baseline analysis is seen.

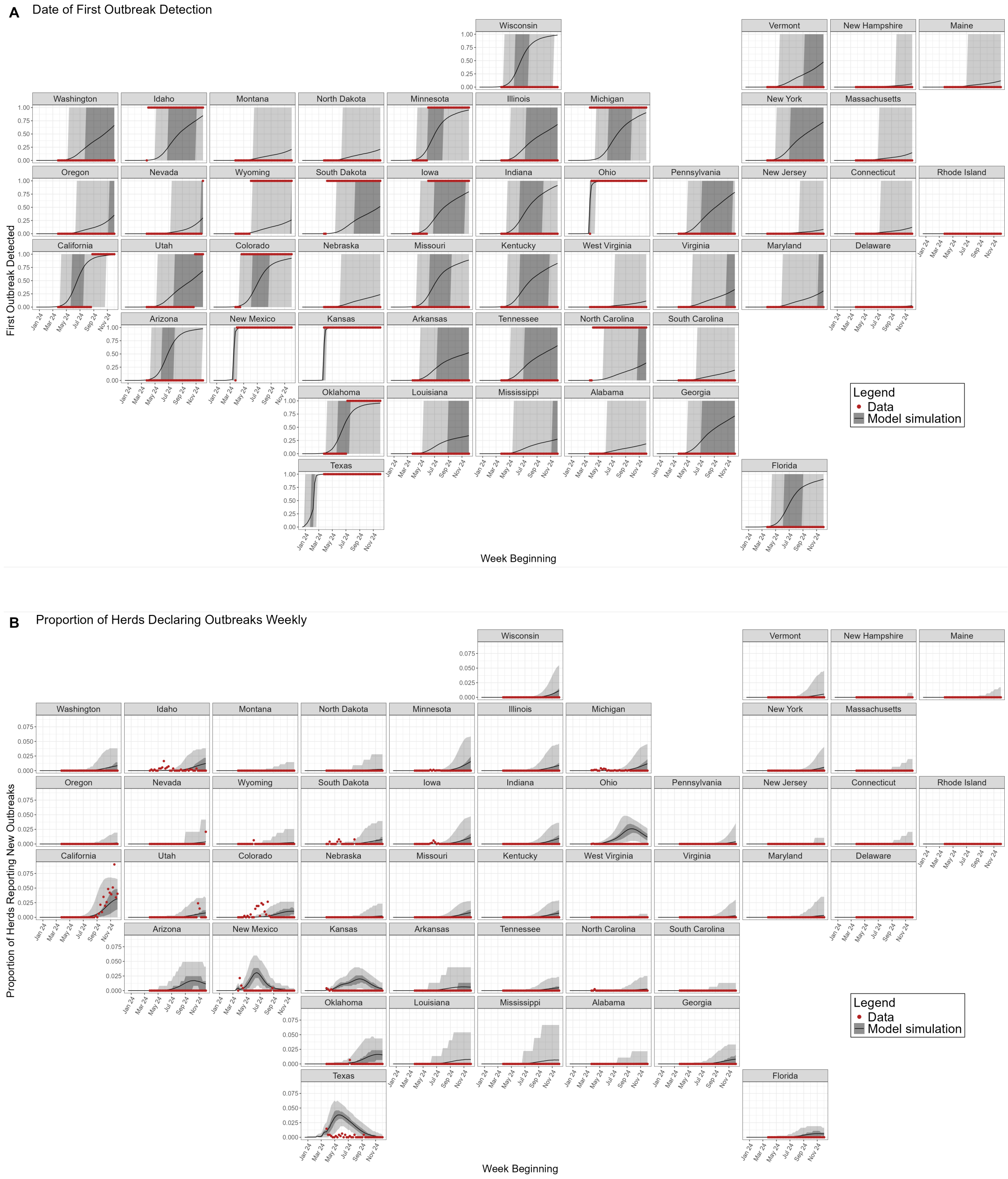

Figure S30: This is a recreation of Figure 2 from the main manuscript when including birth-death processes in the model. **(A)** shows the time at which the first outbreak is detected in a state. **(B)** shows the proportion of herds in each state which report new outbreaks per state each week, accounting for under-reporting. Red points depict real world data. The black line depicts the model mean, the lightly shaded outer grey region depicts the 95% credible interval (95% CrI), while the darker inner grey region depicts the 50% CrI.

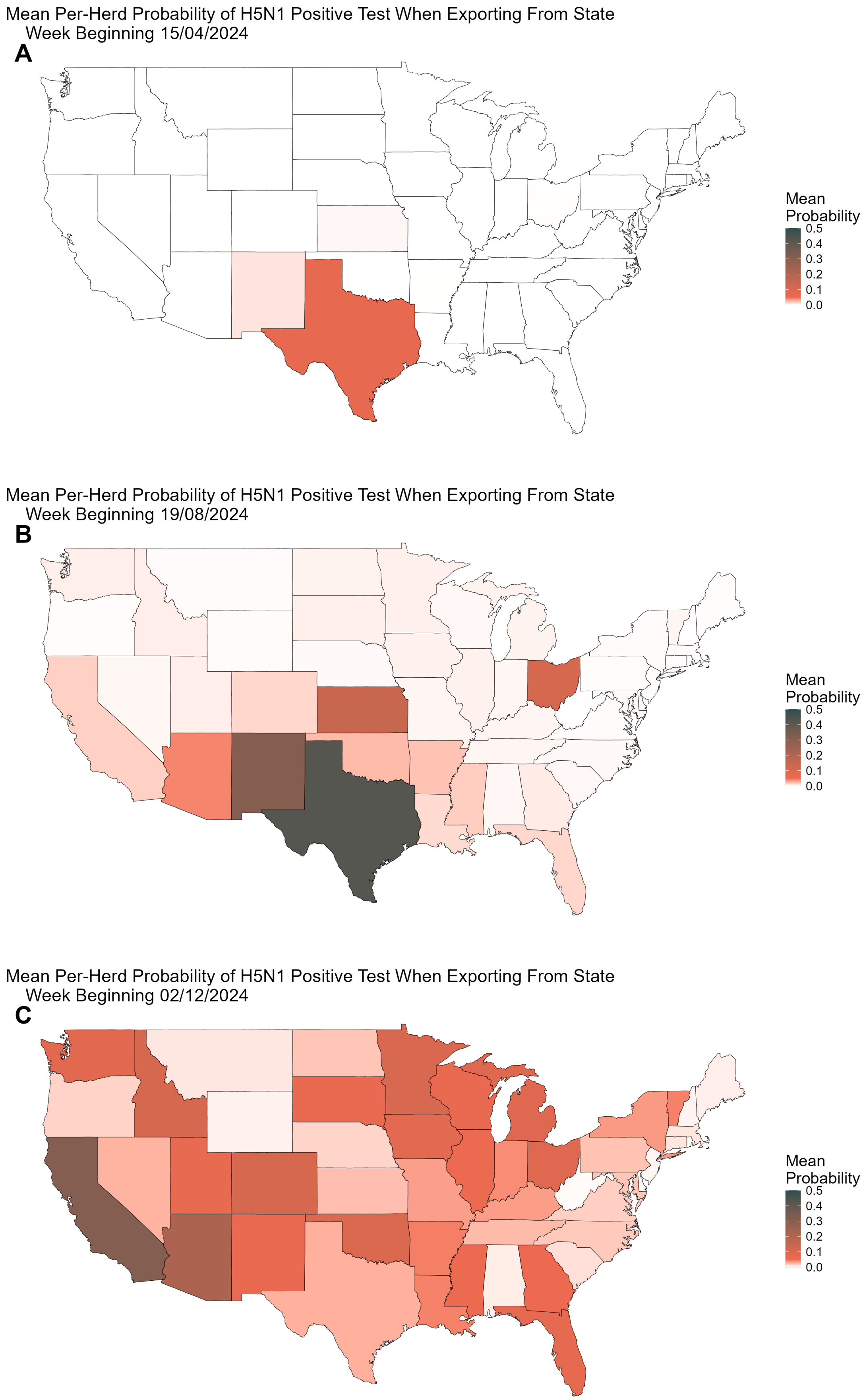

Figure S31: This is a recreation of Figure 4 from the main manuscript when including birth-death processes in the model. When moving cattle inter-state, up to 30 cattle will be tested for H5N1. Above panels show the state average probability of such an export testing positive over time: **(A)** week beginning April 15th 2024, **(B)** week beginning August 19th 2024, and **(C)** week beginning December 2nd 2024.

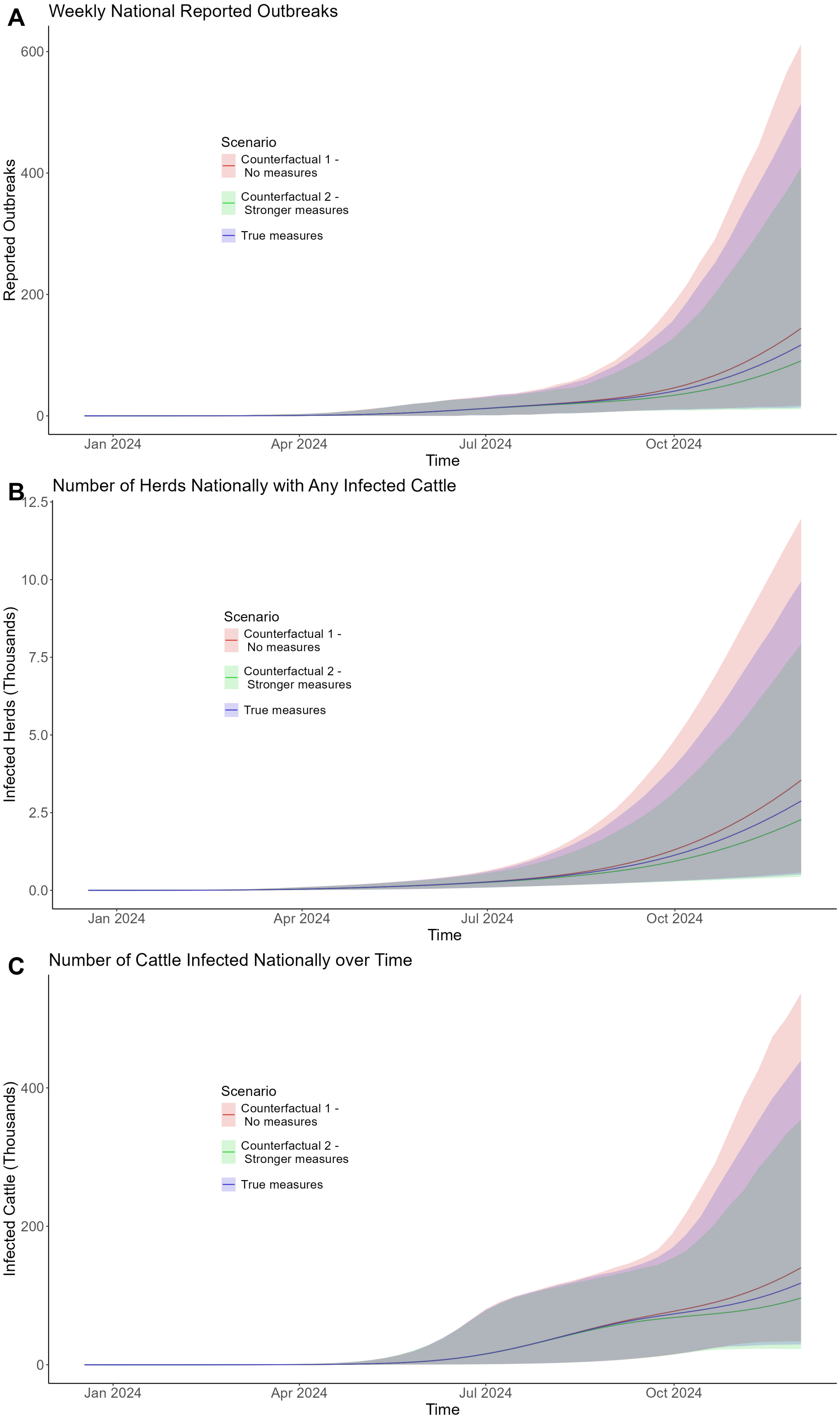

Figure S32: This is a recreation of Figure 5 from the main manuscript when including birth-death processes in the model. **(A)** The number of new reported outbreaks weekly. **(B)** The number of herds nationally with any infected cattle. **(C)** The total number of infected cows nationally over time. Solid lines show simulation mean. Shaded regions show 95% CrI. Blue depicts baseline model assumptions. Red depicts the scenario with no border testing. Green depicts border testing of up to 100 cows, implemented 28 days earlier, on April 1st 2024.

References

[1] United States 2022 Census of Agriculture. Table 11. Cattle and Calves - Inventory and Sales: 2022 and 2017;. Accessed: 2024-12-09. https://www.nass.usda.gov/Publications/AgCensus/2022/Full_Report/Volume_1,_Chapter_2_US_State_Level/st99_2_011_011.pdf.

[2] US Department of Agriculture. Fact Sheet: Highly Pathogenic Avian Influenza H5N1 Genotype B3.13 in Dairy Cattle: National Epidemiologic Brief ;. Accessed: 2024-12-16. https://www.aphis.usda.gov/sites/default/files/hpai-dairy-national-epi-brief.pdf.

[3] Caserta LC, Frye EA, Butt SL, Laverack M, Nooruzzaman M, Covaleda LM, et al. Spillover of highly pathogenic avian influenza H5N1 virus to dairy cattle. Nature. 2024;634:669-76.

[4] Sellman S, Beck-Johnson LM, Hallman C, Miller RS, Bonner KAO, Portacci K, et al. Modeling US cattle movements until the cows come home: Who ships to whom and how many? Computers and Electronics in Agriculture. 2022;203:107483.

[5] Animal and Plant Health Inspection Service. Technical Notes: Clarification to Inquiries Received on April 24 Federal Order;. Accessed: 2024-12-09. https://www.aphis.usda.gov/sites/default/files/tech-notes-clarification-inquiries-rcvd-fo.pdf.

[6] Andrieu C, Doucet A, Holenstein R. Particle markov chain monte carlo methods. Journal of the Royal Statistical Society Series B: Statistical Methodology. 2010;72(3):269-342.

[7] FitzJohn RG, Knock ES, Whittles LK, Perez-Guzman PN, Bhatia S, Guntoro F, et al. Reproducible parallel inference and simulation of stochastic state space models using odin, dust, and mcstate. Wellcome Open Research. 2021;5:288.

[8] Cabezas A, Sanderson M, Lockhart C, Riley K, Hanthorn C. Spatial and network analysis of US livestock movements based on Interstate Certificates of Veterinary Inspection. Preventive Veterinary Medicine. 2021;193:105391.

[9] Vredenberg I, Han R, Mourits M, Hogeveen H, Steeneveld W. An empirical analysis on the longevity of dairy cows in relation to economic herd performance. Frontiers in Veterinary Science. 2021;8:646672.

List of Figures

S1 Mean dairy herd size by state. . . . 3
S2 Weekly probability of a herd exporting cattle. . . . 8
S3 Mean daily dairy cattle exports. . . . 9
S4 Proportion of herd that is exported by state. . . . 11
S5 Movement matrix heatmap. . . . 12
S6 $\frac{\beta}{\gamma}$ chain trajectories. . . . 14
S7 α chain trajectories. . . . 14
S8 σ chain trajectories. . . . 15
S9 γ chain trajectories. . . . 15
S10 A^{asc} chain trajectories. . . . 16
S11 Log-likelihood chain trajectories. . . . 16
S12 $\frac{\beta}{\gamma}$ prior and posterior distributions. . . . 17
S13 α prior and posterior distributions. . . . 17
S14 σ prior and posterior distributions. . . . 18
S15 γ prior and posterior distributions. . . . 18
S16 A^{asc} prior and posterior distributions. . . . 19
S17 Model simulations for number of infected herds. . . . 20
S18 Probability of exported cattle testing positive. . . . 21
S19 Counterfactual scenario with reduced α. . . . 23
S20 Herd populations and Texas import links. . . . 24
S21 Model simulation results with incidence likelihood. . . . 26
S22 Probability of positive border test with incidence likelihood. . . . 27
S23 Counterfactual intervention scenarios with incidence likelihood. . . . 28
S24 Model simulation results with ICVI data. . . . 29
S25 Probability of positive border test with ICVI data. . . . 30
S26 Counterfactual intervention scenarios fit with ICVI data. . . . 31
S27 Model simulation results with simplified ascertainment assumptions. . . . 32
S28 Probability of positive border test with simplified ascertainment assumptions. . . . 33
S29 Counterfactual intervention scenarios fit with simplified ascertainment assumptions. . . . 34
S30 Model simulation results with birth-death processes. . . . 36
S31 Probability of positive border test with birth-death processes. . . . 37
S32 Counterfactual intervention scenarios fit with birth-death processes. . . . 38

List of Tables

S1 US dairy cattle populations . . . 2
S2 Definitions of model parameters . . . 4
S3 Probability of exporting cattle by state . . . 7
S4 Proportion of herd exported by state . . . 10
S5 Model fitting results . . . 13
