# Supplementary material for: A mathematical model of H5N1 influenza transmission in US dairy cattle
Source: Nat Commun. 2025 May 8;16:4308. doi: 10.1038/s41467-025-59554-z (PMC12062519; doi:10.1038/s41467-025-59554-z)
Supplement: Supplementary file 2 — Description of Additional Supplementary Files [file 41467_2025_59554_MOESM2_ESM.pdf]

Description of Additional Supplementary Files

Supplementary Data: Movement Matrix denoting probability of cattle transfer from US state i to stage j
